# Efficient generation of complex vectorial optical fields with metasurfaces

**DOI:** 10.1038/s41377-021-00504-x

**Published:** 2021-03-31

**Authors:** Dongyi Wang, Feifei Liu, Tong Liu, Shulin Sun, Qiong He, Lei Zhou

**Affiliations:** 1 State Key Laboratory of Surface Physics, Key Laboratory of Micro and Nano Photonic Structures (Ministry of Education) and Department of Physics, Fudan University, Shanghai, China; 2 Shanghai Engineering Research Centre of Ultra-Precision Optical Manufacturing, Green Photonics and Department of Optical Science and Engineering, Fudan University, Shanghai, China; 3 Academy for Engineering and Technology, Fudan University, Shanghai, China; 4 Collaborative Innovation Centre of Advanced Microstructures, Nanjing, China

**Keywords:** Nanophotonics and plasmonics, Sub-wavelength optics

## Abstract

Vectorial optical fields (VOFs) exhibiting *arbitrarily designed* wavefronts and polarization distributions are highly desired in photonics. However, current methods to generate them either require complicated setups or exhibit limited functionalities, which is unfavorable for integration-optics applications. Here, we propose a generic approach to efficiently generate arbitrary VOFs based on metasurfaces exhibiting *full-matrix* yet *inhomogeneous* Jones-matrix distributions. We illustrate our strategy with analytical calculations on a model system and an experimental demonstration of a meta-device that can simultaneously deflect light and manipulate its polarization. Based on these benchmark results, we next experimentally demonstrate the generation of a far-field VOF exhibiting both a vortex wavefront and an inhomogeneous polarization distribution. Finally, we design/fabricate a meta-device and experimentally demonstrate that it can generate a complex *near-field* VOF—a cylindrically polarized surface plasmon wave possessing orbital angular momentum—with an efficiency of ~34%. Our results establish an efficient and ultracompact platform for generating arbitrary predesigned VOFs in both the near- and far-fields, which may find many applications in optical manipulation and communications.

## Introduction

Light beams are widely used in photonics applications (e.g., sensing, optical trapping and manipulation). In addition to *homogeneously polarized* light beams, one can also construct *vectorial optical fields* (VOFs) with *tailored* wavefronts and *inhomogeneous* polarization distributions^1^. The added polarization degree of freedom offers VOFs more advantages in applications compared to their scalar-wave counterparts^1,2^. For example, by tailoring the polarization distribution, special VOFs such as flat-top beams and radially polarized beams can be generated, which are highly favored in superresolution microscopy^3–5^, optical manipulation^6^, and many other applications^7–11^. However, conventional methods to generate VOFs require bulky devices and complex setups^12,13^. The key reason is that naturally existing materials only exhibit electrical responses with moderate permittivity at optical frequencies, and thus, conventional devices should be thick enough to ensure appropriate phase accumulation for controlling light. Moreover, to further control the polarization distribution, more devices with different functions are needed, making the final setup bulky and complicated.

Metasurfaces, ultrathin metamaterials consisting of planar microstructures (e.g., meta-atoms) with tailored optical responses arranged in certain global sequences, recently demonstrated extraordinary capabilities to control electromagnetic (EM) waves at deep-subwavelength scales^14–19^. Many fascinating effects have been achieved based on metasurfaces, such as polarization control^20–24^, perfect EM absorption^25–27^, anomalous beam deflection^28–31^, surface wave excitation^32–38^, meta-holography^39,40^, meta-lensing^41,42^, and many others^43–49^. In particular, by designing anisotropic meta-atoms exhibiting distinct reflection/transmission phases ${\Phi}_{ii}$ for waves polarized along two orthogonal directions ($i = x,y$), ultrathin meta-devices that can efficiently control the spin (polarization) properties of EM waves were demonstrated in different frequency regimes^20–24,50,51^. Meanwhile, by designing *inhomogeneous* metasurfaces exhibiting phase profiles ${\Phi}\left( {\vec r} \right)$, wavefronts of light beams can be reconstructed according to Huygens’ law after being scattered by the metasurfaces^29–31^. Recently, certain VOFs were successfully generated using different meta-devices (e.g., multichannel vectorial holography^52^ and polarization-dependent multifocusing^53^). However, the VOFs generated thus far are typically far-field VOFs exhibiting certain limited polarization distributions (e.g., radial or azimuthal linear polarizations)^28,52–64^. Complex VOFs in the near-field or exhibiting generic polarization distributions are, however, rarely generated by metasurfaces.

In this article, we establish a *generic* strategy for designing *ultrathin* meta-devices to *efficiently* generate *arbitrary* VOFs (including both far-field and near-field VOFs) as desired and experimentally demonstrate the concept in the near-infrared (NIR) regime. The key idea is to assume that the meta-device exhibits an inhomogeneous full-matrix Jones matrix, thus possessing control capabilities for both the local spin and global wavefront of a light beam. After elucidating our concept based on model-level analytical calculations, we first perform a benchmark experiment to demonstrate a meta-device that abnormally reflects light and changes its polarization *uniformly*. We next experimentally demonstrate the generation of a far-field VOF exhibiting a vortex wavefront with an inhomogeneous distribution of elliptical polarizations. Finally, we design/fabricate a meta-device and experimentally demonstrate that it can generate a *cylindrically polarized vortex* surface plasmon wave, which is a special near-field VOF exhibiting designed wavefront, polarization distribution, and even orbital angular momentum. All three meta-devices exhibit excellent working performances and ultrabroad bandwidths, and the experimentally measured results are in excellent agreement with numerical simulations and analytical calculations.

## Results

### Concept and model calculations

**Fig. 1:**
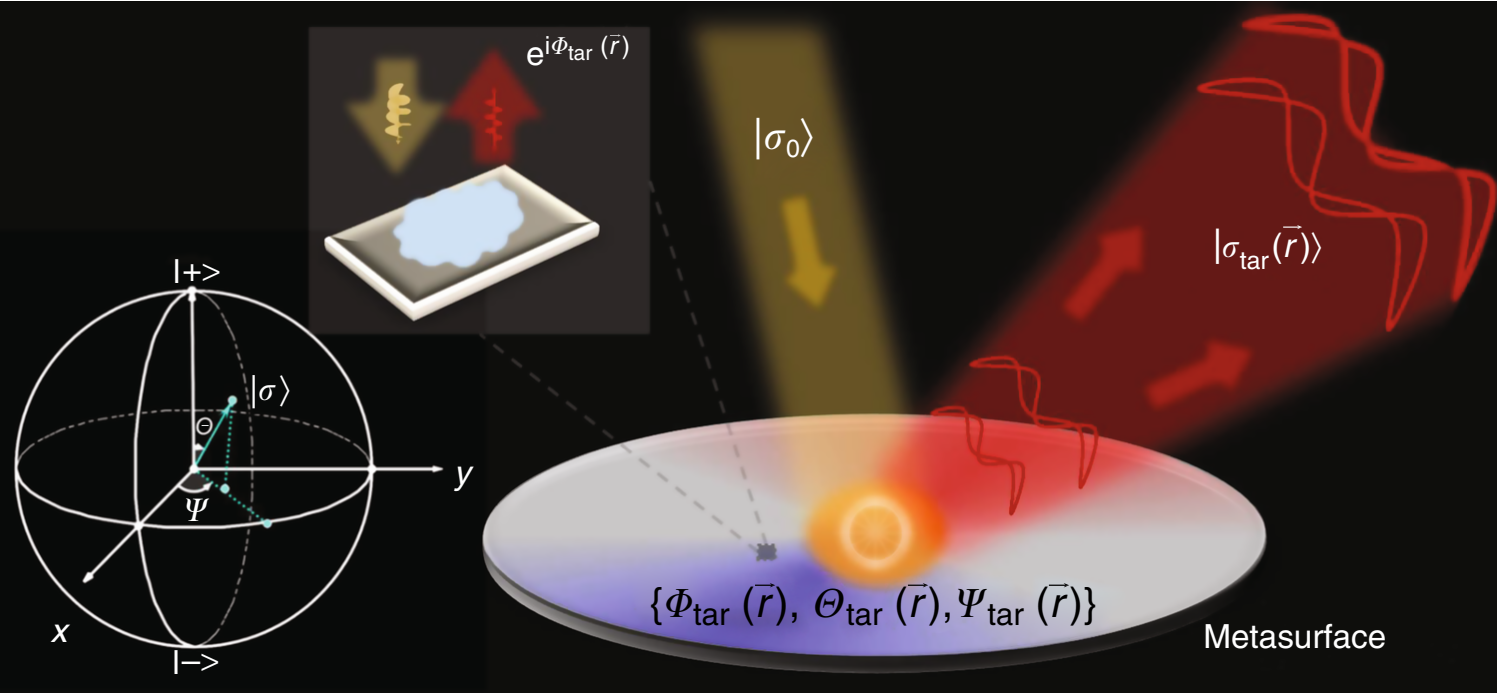
Schematic of generating arbitrary VOFs with metasurfaces. A specifically designed meta-atom can reflect normally incident light with polarization $\left| {\sigma _0} \right\rangle$ (described by a point $({\Theta}_0,{\Psi}_0)$on Poincare’s sphere) to light with a target polarization $\left| {\sigma _{{\mathrm{tar}}}} \right\rangle$ (described by a point $({\Theta}_{{\mathrm{tar}}},{\Psi}_{{\mathrm{tar}}})$ on Poincare’s sphere) with a desired phase ${\Phi}_{{\mathrm{tar}}}$. Using a set of meta-atoms with different ${\Phi}_{{\mathrm{tar}}}$and $\{ {\Theta}_{{\mathrm{tar}}},{\Psi}_{{\mathrm{tar}}}\}$to form a metasurface and illuminating it by normally incident light with polarization $\left| {\sigma _0} \right\rangle$, a VOF can be generated with the wavefront determined by ${\Phi}_{{\mathrm{tar}}}(\vec r)$and the polarization distribution determined by $\{ {\Theta}_{{\mathrm{tar}}}(\vec r),{\Psi}_{{\mathrm{tar}}}(\vec r)\}$on the designed metasurface

As shown in Fig. 1, suppose that a metasurface is illuminated by a normally incident plane wave possessing a uniform polarization represented by a vector $$\left| {\sigma _0} \right\rangle = \left( {\begin{array}{*{20}{c}} {e^{ - i{\Psi}_0/2}\cos ({\Theta}_0/2)} \\ {e^{i{\Psi}_0/2}{\rm{sin}}({\Theta}_0/2)} \end{array}} \right)$$, with $\left( {{\Theta}_0,{\Psi}_0} \right)$ denoting its position on Poincare’s sphere^65^. The following question arises: what properties should the metasurface exhibit if we require the scattered wave to possess a desired wavefront and an inhomogeneous polarization distribution?

To answer this question, we consider a generic reflective metasurface constructed by a set of meta-atoms (see Fig. 1), each exhibiting a different yet generic Jones matrix $${\mathbf{R}}_i{\mathrm{ = }}\left( {\begin{array}{*{20}{c}} {r_{xx}} & {r_{xy}} \\ {r_{yx}} & {r_{yy}} \end{array}} \right) = \left( \begin{array}{l}\left| {r_{xx}} \right|e^{i{\Phi}_{xx}}{\mathrm{ }}\left| {r_{xy}} \right|e^{i{\Phi}_{xy}}\\ \left| {r_{yx}} \right|e^{i{\Phi}_{yx}}{\mathrm{ }}\left| {r_{yy}} \right|e^{i{\Phi}_{yy}}\end{array} \right)$$. Under lossless conditions, energy conservation and time-reversal symmetry impose the following five constraints on Jones-matrix elements (see more details in Sec. 1.1 of the Supplementary Information (SI)):

1 $$\left\{ {\begin{array}{*{20}{c}} {\left| {r_{xx}} \right|^2 + \left| {r_{yx}} \right|^2 = 1,{\mathrm{ }}\left| {r_{xy}} \right|^2 + \left| {r_{yy}} \right|^2 = 1,{\mathrm{ }}\left| {r_{xy}} \right| = \left| {r_{yx}} \right|} \\ {{\Phi}_{xy} = {\Phi}_{yx},{\mathrm{ }}2{\Phi}_{xy} = {\Phi}_{xx} + {\Phi}_{yy} + \left( {2n + 1} \right)\pi } \end{array}} \right.{\mathrm{ }}$$

Therefore, only three degrees of freedom are available to tune the matrix elements in ${\mathbf{R}}_i$. In general, we thus have a matrix function ${\mathbf{R}}(\vec r)$ to model our metasurface, with matrix elements at every local position satisfying Eq. (1). Changing linear-polarization (LP) bases to circular-polarization (CP) bases, we obtain ${\tilde{\mathbf R}}(\vec r) = {\mathrm{S}}{\mathbf{R}}(\vec r){\mathop{\rm{S}}\nolimits} ^{ - 1}$, with $${\mathrm{S}} = \frac{{\sqrt 2 }}{2}\left( {\begin{array}{*{20}{c}} 1 & { - i} \\ 1 & i \end{array}} \right)$$. Given the incident polarization state$\left| {\sigma _0} \right\rangle$, the locally reflected wave (under the lossless condition) can be rewritten as

2 $${\tilde{\mathbf R}}(\vec r)\left| {\sigma _0} \right\rangle = e^{i{\Phi}_{{\mathrm{tar}}}(\vec r)}\left| {\sigma _{{\mathrm{tar}}}(\vec r)} \right\rangle$$

where $$\left| {\sigma _{{\mathrm{tar}}}(\vec r)} \right\rangle = \left( {\begin{array}{*{20}{c}} {e^{ - i{\Psi}_{{\mathrm{tar}}}(\vec r)/2}\cos ({\Theta}_{{\mathrm{tar}}}(\vec r)/2)} \\ {e^{ + i{\Psi}_{{\mathrm{tar}}}(\vec r)/2}\sin ({\Theta}_{{\mathrm{tar}}}(\vec r)/2)} \end{array}} \right)$$ represents a new polarization state corresponding to a point $\left( {{\Theta}_{{\mathrm{tar}}}(\vec r),{\Psi}_{{\mathrm{tar}}}(\vec r)} \right)$ on Poincare’s sphere, and $e^{i{\Phi}_{{\mathrm{tar}}}(\vec r)}$ is a phase factor (see the inset in Fig. 1). We note that both the phase ${\Phi}_{{\mathrm{tar}}}(\vec r)$ and the spin state $\left( {{\Theta}_{{\mathrm{tar}}}(\vec r),{\Psi}_{{\mathrm{tar}}}(\vec r)} \right)$ are dictated by local properties of the matrix ${\tilde{\mathbf R}}(\vec r)$. Equation (2) reveals that by adjusting matrix elements in ${\tilde{\mathbf R}}(\vec r)$ under the constraints of Eq. (1), we can design metasurfaces yielding locally reflected waves with *freely tailored* ${\Phi}_{{\mathrm{tar}}}(\vec r)$ and $\left| {\sigma _{{\mathrm{tar}}}(\vec r)} \right\rangle$, thus realizing any designed VOFs. Strictly speaking, the Jones-matrix description is based on a plane-wave input and is thus applicable to impinging beams in the paraxial approximation. We note that in principle, VOFs thus generated can only exhibit the desired wavefronts and polarization distributions in the vicinity of the metasurface. However, our numerical/experimental results presented in the following sections suggest that the generated VOFs can well preserve their wavefronts and polarization distributions even in the far-field. We find that the designed VOF can exhibit good fidelity as long as the target polarization distribution does not vary strongly in space so that interferences among waves reflected from different local positions do not significantly deteriorate the desired VOF.

We perform analytical calculations to illustrate how the idea works. Consider a model metasurface that, under the illumination of normally incident light with left circular polarization (LCP,$({\Theta}_0,{\Psi}_0) = (0,0)$), can generate locally reflected waves exhibiting the following ${\Phi}_{{\mathrm{tar}}}(\vec r)$ and $\left| {\sigma _{{\mathrm{tar}}}(\vec r)} \right\rangle$:

3 $${\Phi}_{{\mathrm{tar}}}(\vec r) = {\mathbf{2}}{\boldsymbol{\pi}} \frac{\mathbf{r}}{{P_s}} + {\boldsymbol{\varphi}} ;{\boldsymbol{{\Theta}}}_{{\mathrm{tar}}}(\vec {\mathbf{r}}) = \left\{ \begin{array}{*{20}{l}}{\boldsymbol{\varphi}} &{\mathbf{0}} \, \le \, {\boldsymbol{\varphi}} \, < \, {\boldsymbol{\pi}} \\ {\mathbf{2}}{\boldsymbol{\pi}} - {\boldsymbol{\varphi}} &{\boldsymbol{\pi}} \, \le \, {\boldsymbol{\varphi}} \, < \, {\mathbf{2}}{\boldsymbol{\pi}} \end{array} \right.;{\boldsymbol{{\Psi}}}_{{\mathrm{tar}}}(\vec {\mathbf{r}}) = 2{\boldsymbol{\varphi}}$$

**Fig. 2:**
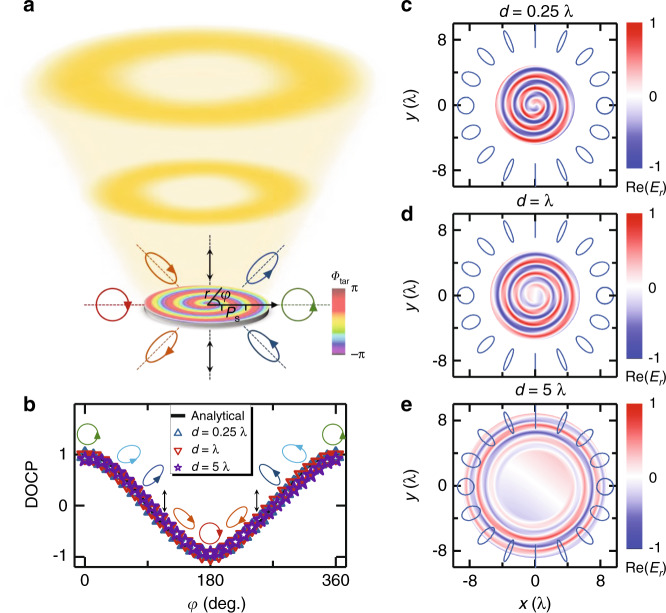
Analytical calculations on a model system for complex VOF generation. **a** Schematic of a light beam scattered by a model metasurface exhibiting designed distributions $\left\{ {{\Phi}_{{\mathrm{tar}}}(\vec r)} \right.,{\mathrm{ }}{\Theta}_{{\mathrm{tar}}}(\vec r),{\mathrm{ }}\left. {{\Psi}_{{\mathrm{tar}}}(\vec r)} \right\}$ given by Eq. (3), generating a divergent vortex beam with spatially dependent polarizations. The color map on the metasurface represents the required ${\Phi}_{{\mathrm{tar}}}(\vec r)$ distribution. **b** DOCP versus polar angle inside the beam computed by the GF approach on *xy*-planes at distance *d* from the metasurface. The solid line represents the analytical predictions by Eq. (3). Ellipses/circles/arrows represent the polarization patterns at different polar angles. **c**–**e** Distributions of ${\Re} \left( {E_r} \right)$ for the reflected beam on *xy*-planes at different distances above the metasurface, computed by the GF approach

Here, $P_s = 1.55\lambda$, with $\lambda$ being the working wavelength, and $\varphi$ and *r* are the polar angle and radius of a vector $\vec r$ in the cylindrical coordinate system (see Fig. 2a). According to Eq. (3), we expect the reflected wave to exhibit a diverging wavefront exhibiting a $\varphi$-dependent polarization distribution and carrying orbital angular momentum (OAM). With Eq. (3) known, we can easily retrieve from Eq. (2) the desired optical properties of the model metasurface:

4 $${\mathbf{R}}(\vec r) = \left\{ {\begin{array}{*{20}{c}} {e^{i2\pi \cdot r/P_s}\left( {\begin{array}{*{20}{c}} {\cos \frac{\varphi }{2} + \frac{i}{2}\left( {\cos \frac{{3\varphi }}{2} - \cos \frac{{5\varphi }}{2}} \right)} & { - i\cos 2\varphi \sin \frac{\varphi }{2}} \\ { - i\cos 2\varphi \sin \frac{\varphi }{2}} & {\cos \frac{\varphi }{2} - \frac{i}{2}\left( {\cos \frac{{3\varphi }}{2} - \cos \frac{{5\varphi }}{2}} \right)} \end{array}} \right){\mathrm{, 0}} \le \varphi\, < \,\pi } \\ {e^{i2\pi \cdot r/P_s}\left( {\begin{array}{*{20}{c}} { - \cos \frac{\varphi }{2} + \frac{i}{2}\left( {\cos \frac{{3\varphi }}{2} - \cos \frac{{5\varphi }}{2}} \right)} & { - i\cos 2\varphi \sin \frac{\varphi }{2}} \\ { - i\cos 2\varphi \sin \frac{\varphi }{2}} & { - \cos \frac{\varphi }{2} - \frac{i}{2}\left( {\cos \frac{{3\varphi }}{2} - \cos \frac{{5\varphi }}{2}} \right)} \end{array}} \right),{\mathrm{ }}\pi \le \varphi \,< \,2\pi } \end{array}} \right.$$

Indeed, Eq. (4) shows that the device should exhibit an *inhomogeneous* and *full-matrix* form of the Jones matrix to control both the wavefront and local polarization properties of a light beam. With the device’s optical properties completely known, we thus employ a Green’s function (GF) approach to analytically study the properties of the light beam reflected by the device under the illumination of normally incident LCP light (more details can be found in Sec. 2 of the Supplementary Information).

Figure 2c–e well reveal the desired VOF properties of the reflected beam, consistent with our theoretical expectations. The patterns of ${\Re} \left( {E_r} \right)$ computed on different planes suggest that the wavefront of the reflected beam gradually diverges as it leaves the metasurface, exhibiting well-defined vortex features. Meanwhile, Fig. 2b depicts how the calculated local degree-of-circular polarization (DOCP), defined as ${\mathrm{DOCP}} = \left( {\left| {E_r - iE_\varphi } \right|^2 - \left| {E_r + iE_\varphi } \right|^2} \right)/\left( {\left| {E_r - iE_\varphi } \right|^2 \, + \, \left| {E_r + iE_\varphi } \right|^2} \right)$, varies against the azimuthal angle $\varphi$ inside the reflected beam on different *xy*-planes, where ${\mathrm{\{ }}E_r,E_\varphi ,E_{z^{\prime}}{\mathrm{\} }}$ are electric-field components measured in the local cylindrical coordinate systems where the local *z*′ axis is parallel to the propagation direction. Calculations of DOCP distributions are performed on the corresponding circles where the electric-field amplitude reaches its maximum on each observation plane at different distances. We find that the local polarization is generally elliptical, but with both the ellipticity and polar angle varying continuously as functions of $\varphi$, exactly following the analytical predictions (solid lines in Fig. 2b) given by Eq. (3). In addition, the polarization distribution is well preserved during propagation of the reflected light beam, showing the robustness of the generated VOF. We note that the generated VOF suffers from the diffraction effect, and thus, its donut-shaped wavefront becomes less perfect in the far-field (see Fig. S2 in the Supplementary Information). However, our calculations reveal that the power taken away by the diffraction fringes is quite small (e.g., less than 12% in Fig. S2 in the Supplementary Information). If we reduce the divergence angle of the generated VOF from 40° to 15°, we find that the working efficiency can be further improved (94%) since the diffraction effect becomes weaker.

### Design strategy and characterization of meta-atoms

**Fig. 3:**
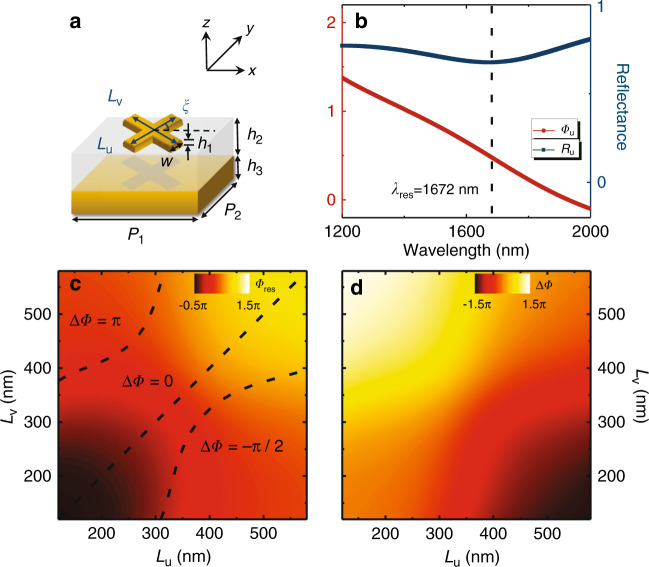
Meta-atom design. **a** Schematic of the designed MIM meta-atoms with varying $L_u$ and $L_v$ and fixed $h_1 = 30\,{\mathrm{nm}},\,h_2 = 180\,{\mathrm{nm}},\,h_3 = 125\,{\mathrm{nm}}$, $w = 120\,{\mathrm{nm}}$, and $P_1 = P_2 = 600\,{\mathrm{nm}}$. **b** FDTD-simulated spectra of reflectance $R_u$ and phase ${\Phi}_u$ for the meta-atom with $L_u = 367\,{\mathrm{nm}}$ and $L_v = 120\,{\mathrm{nm}}$ under $\vec E//\hat u$ polarized incident light. Phase diagrams of **c** ${\Phi}_{{\mathrm{res}}}$ and **d** ${\Delta}{\Phi}$ versus $L_u$ and $L_v$ obtained by FDTD simulations at a wavelength of 1550 nm

We now describe our strategy to construct meta-devices exhibiting designed *inhomogeneous full-matrix* Jones matrices, starting from a search for a set of appropriate reflective meta-atoms. As shown in Fig. 3a, our basic *meta-atom* is in a metal-insulator-metal (MIM) configuration, consisting of a gold (Au) resonator and a continuous 125 nm-thick Au film separated by a 180 nm-thick SiO_2_ dielectric spacer. The top resonator is a metallic cross formed by two bars of different lengths (denoted $L_u$ and $L_v$), with the principal axes rotated by an angle *ξ* with respect to those in the laboratory system. Such meta-atoms exhibit three *independent* geometry-tuning parameters ($L_u$, $L_v$ and *ξ*), which can help us design reflective meta-atoms with desired properties dictated by the three parameters {${\Phi}_{{\mathrm{tar}}},{\Theta}_{{\mathrm{tar}}},{\Psi}_{{\mathrm{tar}}}$} under the constraints in Eq. (1).

Consider first the properties of these meta-atoms in their $\{ \hat u,\hat v\}$ coordinate systems. Such MIM meta-atoms support two magnetic resonances for light polarization along the two principal axes $\{ \hat u,\hat v\}$, with resonance frequencies dictated mainly by the lengths of the two bars ($L_u$ and $L_v$). Considering the symmetry and the back mirror in the meta-atom, we find that the Jones matrix of such a meta-atom represented in the $\{ \hat u,\hat v\}$ system can be generally written as $${\mathbf{R}}(0) = \left( {\begin{array}{*{20}{c}} {|r_{uu}{\mathrm{|e}}^{i{\Phi}_u}} & 0 \\ 0 & {|r_{vv}{\mathrm{|e}}^{i{\Phi}_v}} \end{array}} \right)$$. In the ideal lossless condition, we have $|r_{uu}|{\mathrm{ = }}|r_{vv}| \equiv 1$. With losses in realistic materials taken into account, $|r_{uu}|$ and $|r_{vv}|$ are no longer exactly 1 but can still be quite close to 1 via careful design (Fig. 3b). Meanwhile, the reflection phases (${\Phi}_u,{\Phi}_v$) vary sensitively as the frequency passes across two resonances, covering nearly a $2\pi$ range (see Fig. 3b and Sec. 3 in the SI). Therefore, at a fixed frequency, varying $L_u$ and $L_v$ can dramatically change the two resonance frequencies, thus tuning ${\Phi}_u$ and ${\Phi}_v$ freely inside the allowed phase range. We define two new independent parameters ${\Phi}_{{\mathrm{res}}} = \left( {{\Phi}_u + {\Phi}_v} \right)/2 - \pi /4$ and ${\Delta}{\Phi} = {\Phi}_v - {\Phi}_u$, which exhibit clear physical meanings of the averaged resonance phase and cross-polarization phase difference. These two intermediate parameters (i.e., ${\Phi}_{{\mathrm{res}}}$ and ${\Delta}{\Phi}$), instead of the two original phases ${\Phi}_u$ and ${\Phi}_v$, play key roles in designing our metasurfaces. Figure 3c–d depict how the ${\Phi}_{{\mathrm{res}}}$ and ${\Delta}{\Phi}$ of our meta-atoms vary against $L_u$ and $L_v$, calculated at a wavelength of 1550 nm with other geometric parameters of the meta-atoms fixed.

Considering the rotation operation further, the Jones matrix of our meta-atom in CP bases is generally written as

5 $${\tilde{\mathbf R}} = {\mathrm{SM}}(\xi ){\mathbf{R}}({\Phi}_{{\mathrm{res}}},{\Delta}{\Phi}){\mathrm{M}}^{ - 1}(\xi ){\mathrm{S}}^{ - 1}$$

where $${\mathrm{M}}(\xi ){\mathrm{ = }}\left( {\begin{array}{*{20}{c}} {\cos \xi } & { - \sin \xi } \\ {\sin \xi } & {\cos \xi } \end{array}} \right)$$ is responsible for the rotation operation. Obviously, the matrix elements in ${\tilde{\mathbf R}}$ are now determined by three parameters: ${\tilde{\mathbf R}}(\xi ,{\Phi}_{{\mathrm{res}}},{\Delta}{\Phi})$. We can then solve the matrix equation

6 $${\tilde{\mathbf R}}(\xi ,{\Phi}_{{\mathrm{res}}},{\Delta}{\Phi})\left| {\sigma _0} \right\rangle = \exp (i{\Phi}_{{\mathrm{tar}}})\left| {\sigma _{{\mathrm{tar}}}} \right\rangle {\mathrm{ }}$$

to determine the relations between ${\mathrm{\{ }}{\Phi}_{{\mathrm{tar}}},{\Theta}_{{\mathrm{tar}}},{\Psi}_{{\mathrm{tar}}}{\mathrm{\} }}$ and $\{ \xi ,{\Phi}_{{\mathrm{res}}},{\Delta}{\Phi}\}$, with $\left( {{\Theta}_0,{\Psi}_0} \right)$ given as the initial condition.

In the special case of LCP incidence (i.e., ${\Theta}_0 = {\Psi}_0 = 0$), we find the following simple analytical solutions:

7 $${\Phi}_{{\mathrm{tar}}} = {\Phi}_{{\mathrm{res}}} + \xi; \quad {\Theta}_{{\mathrm{tar}}} = {\Delta}{\Phi}; \quad {\Psi}_{{\mathrm{tar}}} = 2\xi - \frac{\pi }{2}{\mathrm{ }}$$

for ${\Delta}{\Phi} \ne \pm \pi$. The solution for the special case of ${\Delta}{\Phi} = \pm \!\pi$ is given in Sec. 1.2 of the SI. Equation (7) exhibits clear physical meanings. After light is reflected by our meta-atom, its polarization changes from LCP to an elliptical polarization with ellipticity determined by ${\Delta}{\Phi}$ and polar angle determined by $\xi$ (apart from a trivial constant $- \pi /2$). Meanwhile, the target phase ${\Phi}_{{\mathrm{tar}}}$ of the reflected wave is dictated collectively by ${\Phi}_{{\mathrm{res}}}$ and $\xi$, with the latter bearing the same physics as the Berry phase^66–71^. Such a Berry-like phase is $\xi$ rather than $2\xi$ in the cases of ${\Delta}{\Phi} \;\ne\; \pm\! \pi$ simply because the polarization eigenstates explicitly depend on $\xi$, which contains additional phase factors $e^{ \pm i\xi }$ (see Sec. 1.2 in the SI). For general cases other than LCP incidence, however, the relations between ${\mathrm{\{ }}{\Phi}_{{\mathrm{tar}}},{\Theta}_{{\mathrm{tar}}},{\Psi}_{{\mathrm{tar}}}{\mathrm{\} }}$ and ${\mathrm{\{ }}\xi ,{\Phi}_{{\mathrm{res}}},{\Delta}{\Phi}{\mathrm{\} }}$ are complicated (see Sec. 1.2 of the Supplementary Information) but can always be numerically obtained. With the required ${\mathrm{\{ }}\xi ,{\Phi}_{{\mathrm{res}}},{\Delta}{\Phi}{\mathrm{\} }}$ for all our meta-atoms known, we can then retrieve from Fig. 3 their geometrical parameters $\left( {L_u,L_v} \right)$ and rotation angles $\xi$ and finally design the metasurface by putting them in appropriate positions.

**Fig. 4:**
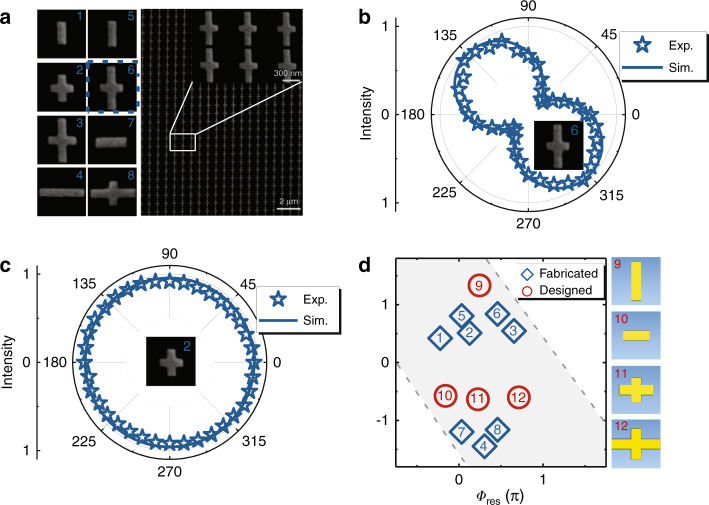
Experimental/numerical characterization of designed meta-atoms. **a** Scanning electron microscopy (SEM) images of 8 experimentally characterized meta-atoms and a periodic metasurface containing an array of the 6^th^ meta-atom with geometrical parameters $L_u = 303{\mathrm{nm}}$, $L_v = 564{\mathrm{nm}}$, and $P_u = P_v = 600{\mathrm{nm}}$. Polarized-filtered intensity patterns of light reflected by periodic metasurfaces containing **b** the 6^th^ meta-atom and **c** the 2^nd^ meta-atom under the illumination of normally incident LP light at 1550 nm, obtained by experiments (stars) and simulations (line). **d** Locations of fabricated and designed meta-atoms on the ${\Delta}{\Phi} \sim {\Phi}_{{\mathrm{res}}}$ phase diagram obtained by numerical simulations and experimental results at 1550 nm

As an illustration, we design 12 meta-atoms (working at 1550 nm) that can occupy a wide space in the full ${\Phi}_{{\mathrm{res}}} \sim {\Delta}{\Phi}$ phase diagram (Fig. 4d) and characterize their optical properties via experiments and simulations. Figure 4a depicts a fabricated sample consisting of a periodic array of the 6^th^ meta-atom, which is numerically found to be located at the position of $({\Delta}{\Phi} = \pi ,{\mathrm{ }}{\Phi}_{{\mathrm{res}}} = 0.47\pi )$ in the phase diagram (Fig. 4d). To experimentally characterize the optical properties of the meta-atom, we illuminate the sample by normally incident LP light with an **E** vector lying at 45° with respect to the *u* axis and measure the signals reflected by the sample filtered by a 360° rotatable linear polarizer placed in front of our detector (see Sec. 8 in the Supplementary Information for the experimental setup). The inset in Fig. 4b compares the measured and simulated polarizer-filtered power patterns at a wavelength of 1550 nm. The obtained “8”-shaped patterns with symmetric axes lying along the *φ* = 135° direction are strong evidence that the meta-atom behaves as a half-wave plate with ${\Delta}{\Phi} = \pi$. Similarly, we also experimentally characterize the optical properties of the 2^nd^ meta-atom, which is numerically identified to be located at $({\Delta}{\Phi} = \pi /2,{\mathrm{ }}{\Phi}_{{\mathrm{res}}} = 0.13\pi )$in the phase diagram (Fig. 4d). Figure 4c depicts the measured/simulated polarizer-filtered power patterns of light reflected by this sample at a wavelength of 1550 nm. The obtained circular patterns well demonstrate that the reflected light exhibits CP and that the meta-atom functions as a quarter-wave plate (i.e., ${\Delta}{\Phi} = \pi /2$), consistent with our theoretical predictions. Unfortunately, the ${\Phi}_{{\mathrm{res}}}$ values of these meta-atoms are difficult to obtain via this type of experiment, and thus, we have to rely on numerical simulations to determine them. Both experiments and simulations demonstrate that these meta-atoms exhibit very high efficiencies (with $\left| {r_{uu}} \right|$ and $\left| {r_{vv}} \right|$ larger than 0.87) and relatively wide working bandwidths (see Fig. S4 in the Supplementary Information).

Before closing this subsection, we discuss an important property of the phase diagram. Due to the invariances in adding $\pm 2\pi$ to ${\Phi}_u$ and/or ${\Phi}_v$, we find that $\left( {{\Phi}_{{\mathrm{res}}}^\prime = {\Phi}_{{\mathrm{res}}} \pm \pi ,\,{\Delta}{\Phi}^{\prime} = {\Delta}{\Phi} \pm 2\pi } \right)$ actually represents the same point as $\left( {{\Phi}_{{\mathrm{res}}},{\mathrm{ }}{\Delta}{\Phi}} \right)$ in the phase diagram. As a result, we only need to consider the shaded region sandwiched between the two dashed lines in the phase diagram (Fig. 4d). We note that the designed 12 meta-atoms still do not fully occupy the whole shaded region, but we can easily design more meta-atoms to fill the unoccupied space. With all necessary meta-atoms designed, we can easily realize any target metasurface for controlling both the wavefront and local polarization distribution of light.

### Benchmark test: an anomalous-reflection meta-wave plate

As a benchmark test, we now use the meta-atoms designed in the last subsection to construct a meta-device that efficiently reflects normally incident LP light to a designed angle with polarization changed to the cross direction. Such a benchmark test can well demonstrate the feasibility of our general strategy to design meta-devices for manipulating the wavefront and polarization of a light beam simultaneously. To achieve this goal, we require our meta-device to exhibit the following characteristic functions:

8 $${\Phi}_{{\mathrm{tar}}}(\vec r) = \zeta _xx;{\mathrm{ }}{\Theta}_{{\mathrm{tar}}}(\vec r) = \frac{\pi }{2};{\mathrm{ }}{\Psi}_{{\mathrm{tar}}}(\vec r) = \frac{{3\pi }}{2}$$

with the incident polarization being LP with ${\Theta}_0 = {\Psi}_0 = \pi /2$. Here, $\zeta _x = 2\pi /1.55\lambda$ ($\lambda = 1550$nm) is the designed phase gradient to generate the anomalous reflection, thus changing the wavefront of light. Meanwhile, the designed parameters $\left( {{\Theta}_{{\mathrm{tar}}}(\vec r),{\mathrm{ }}{\Psi}_{{\mathrm{tar}}}(\vec r)} \right)$ ensure that the reflected light takes a homogeneous distribution of LP but with direction perpendicular to that of the incident polarization.

We now design the meta-device following the general strategy presented in the last subsection. Putting Eq. (8) and ${\Theta}_0 = {\Psi}_0 = \pi /2$ into Eq. (6), we find that the designed meta-device should exhibit $\xi (x) \equiv 0,{\mathrm{ }}{\Phi}_{{\mathrm{res}}}(x) = \zeta _xx - 3\pi /4,{\mathrm{ }}{\Delta}{\Phi}(x) \equiv \pi$. These requirements assist us in finding 4 meta-atoms (labeled No. 5–8) in Fig. 4d, which possess linearly increasing ${\Phi}_{{\mathrm{res}}}$ and a fixed value of ${\Delta}{\Phi} = \pi$. By putting these meta-atoms into a supercell with adjacent distance $P_x = 1.55\lambda$ and then repeating these supercells (see schematic in Fig. S6), we finally construct the meta-device with the desired optical properties.

**Fig. 5:**
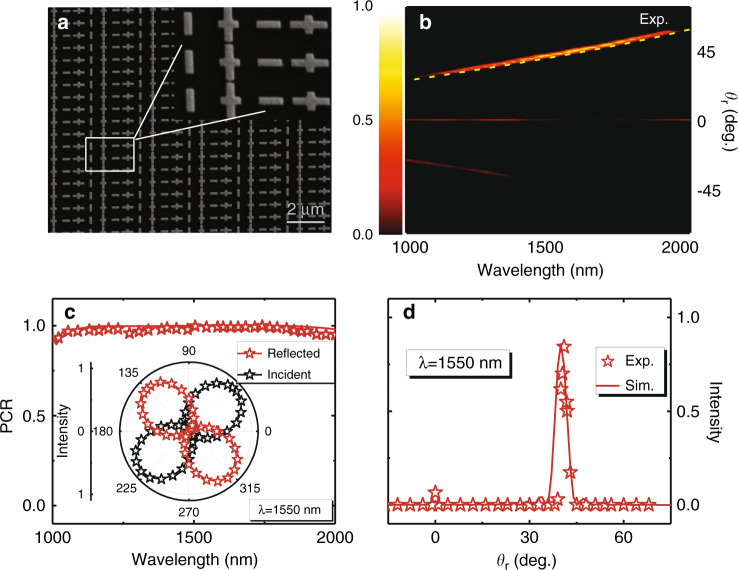
Experimental demonstration of an anomalous-reflection half-wave plate. **a** SEM image of the fabricated metasurface. **b** Measured normalized far-field intensity versus receiving angle and wavelength of light reflected by the metasurface when illuminated by normally incident LP light with an **E**-field polarized along an angle of $45^ \circ$ with respect to the *x*-axis. The yellow dashed line represents theoretical predictions by the generalized Snell’s law. **c** Measured (star) and FDTD-simulated (line) PCR spectra of the metasurface. Inset: measured and simulated polarizer-filtered intensity patterns of light reflected by our meta-device and an Au mirror, both under illumination by normally incident LP light at 1550 nm. **d** Measured (star) and simulated (line) angular distributions of the normalized intensity of light reflected by our meta-device under the same illumination as in **b** and **c** at the wavelength of 1550 nm

We fabricate the sample (see Fig. 5a for its SEM image) and experimentally characterize its scattering properties at telecom wavelengths. Illuminating our sample by normally incident light with the designed linear polarization, we employ our homemade macroscopic angle-resolved spectrometer to measure the intensity of reflected light at different receiving angles (see Sec. 8 in the SI for the experimental setup). As shown in Fig. 5b, within the working band (1150–1850 nm), most of the incident energy is *anomalously reflected* to the designed angle, well matching that (dashed line in Fig. 5b) predicted by the generalized Snell’s law $\theta _r = \sin ^{ - 1}(\zeta _x/k_0)$^29–31^ (see numerical simulation results in Fig. S7 in the Supplementary Information). Figure 5d shows how the reflected signal, normalized against that reflected from a metallic mirror, varies against the receiving angle at a wavelength of 1550 nm. The absolute working efficiency of such anomalous reflection is found to be as high as 85%. We next characterize the polarization property of the anomalously reflected light beam based on measurements similar to those in the last subsection. At the working wavelength, the reflected light turns into LP light with an **E**-field perpendicular to that of the incident light, demonstrated by the “8”-shaped patterns obtained experimentally and numerically (inset in Fig. 5c). Furthermore, Fig. 5c shows that the polarization conversion ratio (PCR, the power ratio of the cross-polarized component of the reflected light) of our meta-device exceeds 90% inside an ultrabroad wavelength range (1000–2000 nm).

### Generation of a far-field VOF: a vortex beam with an inhomogeneous polarization distribution

**Fig. 6:**
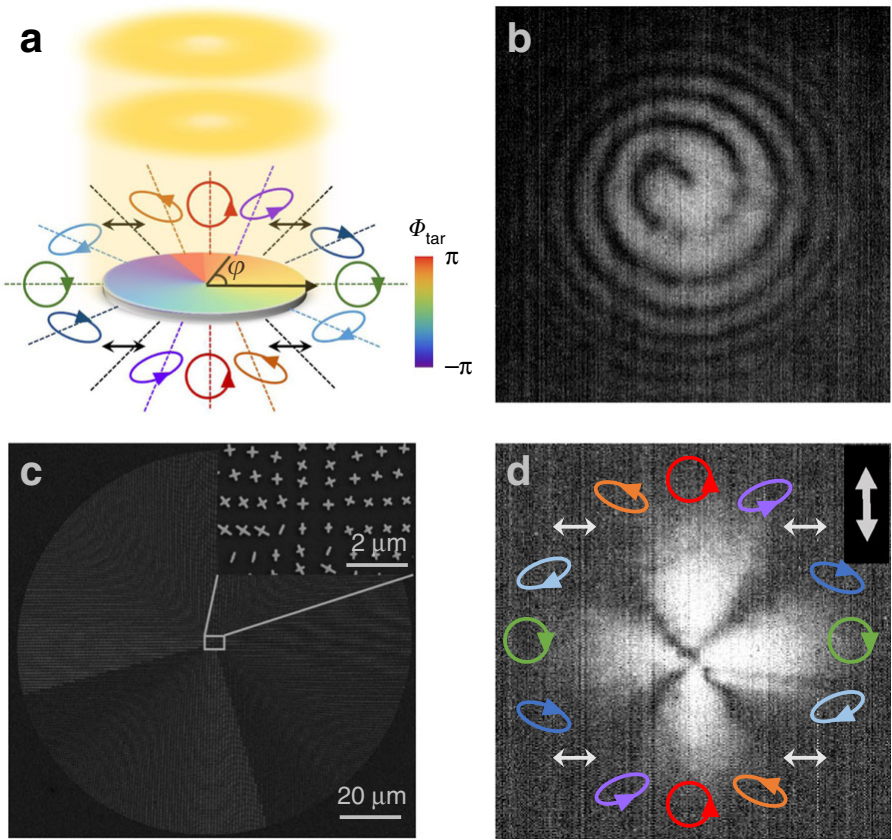
Schematic, sample picture, and characterization of the vortex beam with varying ellipticity. **a** Schematic of our proposed meta-device to generate a vortex beam with varying ellipticity. **b** Interference pattern between the transmitted light and a spherical wave, recorded by our CCD. **c** SEM image of the fabricated sample. **d** Optical image recorded by our CCD for the generated VOF after passing through a linear polarizer with a tilt angle of 90°

We continue to illustrate the powerfulness of our strategy by generating a far-field VOF with both a tailored wavefront and an inhomogeneous polarization distribution. Without loss of generality, the VOF is assumed to be a vortex beam exhibiting spatially varying elliptical polarizations (see Fig. 6a). Based on the theory described above, our meta-device should exhibit the following properties:

9 $${\Phi}_{{\mathrm{tar}}}(\vec r) = \varphi + \frac{\pi }{4};{\mathrm{ }}{\Theta}_{{\mathrm{tar}}}(\vec r) = \pi - 2\varphi ;{\mathrm{ }}{\Psi}_{{\mathrm{tar}}}(\vec r) = 2\varphi - \frac{\pi }{2}$$

under LP plane-wave incidence (i.e., ${\Theta}_0 = \pi /2,{\Psi}_0 = 0$). Obviously, the $\varphi$ term in ${\Phi}_{{\mathrm{tar}}}(\vec r)$offers the reflected beam OAM with topological charge $l = 1$, while the $\{ {\Theta}_{{\mathrm{tar}}}(\vec r),{\Psi}_{{\mathrm{tar}}}(\vec r)\}$ functions precisely describe how the local polarization inside the reflected beam varies against $\varphi$. In particular, Eq. (9) indicates that both the polar angle (dictated by ${\Psi}_{{\mathrm{tar}}}$) and the ellipticity (dictated by ${\Theta}_{{\mathrm{tar}}}$) of the polarization state sensitively depend on $\varphi$, resulting in a fascinating polarization distribution, as shown in Fig. 6a. Apparently, such a VOF well distinguishes itself from those previously realized, which typically exhibit simple cylindrical polarizations (e.g., radial or azimuthal linear polarizations)^56,57,60,61^.

Putting Eq. (9) into Eq. (6), we retrieve the required Jones-matrix distribution of our metasurface $${\mathbf{R}}(\vec r) = \frac{{ie^{i\varphi }}}{{\sqrt 2 }}\left( {\begin{array}{*{20}{c}} { - i + \sin 2\varphi } & { - \!\cos 2\varphi } \\ { - \!\cos 2\varphi } & { - i - \sin 2\varphi } \end{array}} \right)$$, which indeed exhibits an inhomogeneous full-matrix form. Following the general design strategy discussed in Sec. 2.2, we find that our meta-device should exhibit the following $\{ \xi (\vec r),{\Phi}_{{\mathrm{res}}}(\vec r),{\Delta}{\Phi}(\vec r)\}$ distributions:

10 $$\xi (\vec r) = \varphi + \frac{\pi }{4},{\mathrm{ }}{\Phi}_{{\mathrm{res}}}(\vec r) = \varphi - \frac{\pi }{4},{\mathrm{ }}{\Delta}{\Phi}(\vec r) \equiv \frac{\pi }{2}$$

Equation (10) tells us that all meta-atoms inside such a meta-device should function as quarter-wave plates (i.e., ${\Delta}{\Phi}(\vec r) \equiv \pi /2$), though exhibiting different resonance phases ${\Phi}_{{\mathrm{res}}}$ and rotation angles $\xi$. We then retrieve the geometrical parameters ${\mathrm{\{ }}L_u\left( {\vec r} \right),L_v\left( {\vec r} \right){\mathrm{,}}\xi \left( {\vec r} \right){\mathrm{\} }}$ of all meta-atoms based on Eq. (10) and Fig. 4d and finally design and fabricate the meta-device (see Fig. 6c).

We experimentally characterize the performance of the fabricated meta-device. Illuminating the meta-device by normally incident LP light at a wavelength of 1550 nm, we employ a homemade Michelson interferometer to perform interference measurements to reveal the OAM features of the reflected beam. Figure 6b depicts the intensity pattern obtained by interfering the generated VOF with a spherical wave. The 1^st^-order spiral pattern shown in Fig. 6b clearly reveals that the generated VOF exhibits OAM with l = +1, as expected. We further examine the polarization distribution of the generated VOF. Placing a rotatable linear polarizer in front of the charge-coupled device (CCD), we find that the measured polarizer-filtered intensity patterns are highly inhomogeneous and completely different as the polarizer is rotated to different angles (see Supplementary Movie M1 in the Supplementary Information). These features are already strong evidence that the generated VOF indeed exhibits an inhomogeneous polarization distribution. As an illustration, we depict in Fig. 6d the measured pattern as the polarizer is rotated to the vertical direction. Four intensity zeros appear in Fig. 6d, indicating that the local polarization states are LPs at these particular angles, consistent with the designed inhomogeneous polarization distribution. At azimuthal angles other than these four special angles, regardless of how we rotate the polarizer, we can never obtain intensity zeros, indicating that the local polarizations must be elliptical and circular (see Supplementary Movie M1 in the Supplementary Information).

### Generation of a near-field VOF: a vectorial vortex surface plasmon wave

**Fig. 7:**
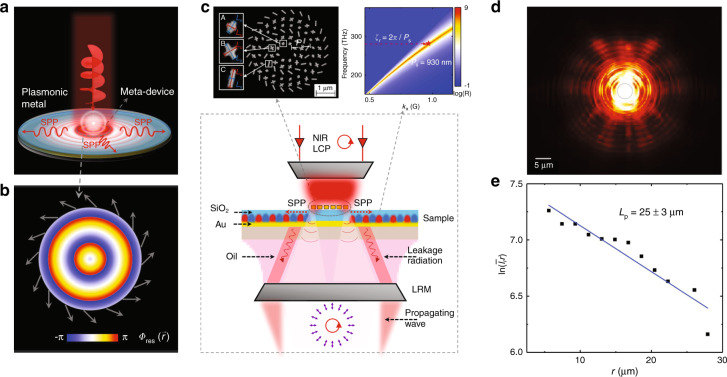
Concept, sample, experimental setup and partial characterization of the vectorial vortex surface plasmon coupler. **a** Schematic of our proposed meta-device to generate a cylindrically polarized vortex SPP beam. **b** Color map representing the designed ${\Phi}_{{\mathrm{res}}}(\vec r)$ distribution on the meta-device with surrounding arrows denoting the directions of $\xi$. **c** Schematic of our leakage radiation microscopy (LRM) setup. The upper left inset is an SEM image of the fabricated sample utilizing three different meta-atoms A, B and C (see all geometric parameters in Sec. 7.1 of the SI), and the upper right inset illustrates the dispersion of SPPs supported by the guiding-out plasmonic metal. **d** LRM-recorded image of the surface wave generated by our meta-device, which is illuminated by normally incident LCP light at a wavelength of 1064 nm. **e** Measured $\ln (\bar I_rr)$ versus r (solid squares), fitted with a linear relation (solid line) with the slope determining the SPP propagation length

While in last subsection, we have demonstrated the generation of a VOF, the generated beam still corresponds to a far-field VOF. In this subsection, we illustrate the full power of our proposed strategy to experimentally demonstrate a meta-device that can generate a special near-field VOF, that is, a cylindrically polarized vortex surface plasmon wave. To achieve this goal, we require the meta-device to generate reflected waves exhibiting the following properties:

11 $${\Phi}_{{\mathrm{tar}}}(\vec r) = \zeta _rr + \varphi ;{\mathrm{ }}{\Theta}_{{\mathrm{tar}}}(\vec r) = \frac{\pi }{2};{\mathrm{ }}{\Psi}_{{\mathrm{tar}}}(\vec r) = 2\varphi$$

with the incident polarization set as LCP (${\Theta}_0 = {\Psi}_0 = 0$). Here, we choose the working wavelength as $\lambda = 1064{\mathrm{ nm}}$ to fit our experimental characterizations. We set $\zeta _r = 2\pi /0.87\lambda \, > \, k_0$ so that the device can convert normally incident light to a surface wave (SW)^32–38^. Meanwhile, the generated SW possesses OAM with topological charge $l = + 1$ due to the presence of the term $\varphi$ in ${\Phi}_{{\mathrm{tar}}}(\vec r)$. Finally, the polarization state at point $\vec r$ inside the ‘reflected wave’ should be generally LP (${\Theta}_{{\mathrm{tar}}}(\vec r) = \pi /2$), but with the polarization angle parallel to the radial direction (${\Psi}_{{\mathrm{tar}}}(\vec r) = 2\varphi$). Collecting all the above information, we expect from Eq. (11) that the generated VOF must be a cylindrically polarized vortex surface wave, as schematically shown in Fig. 7a.

We now design the meta-device based on Eq. (11). Putting Eq. (11) into Eq. (6), we can easily retrieve the desired Jones matrix of our metasurface as $${\mathbf{R}}(\vec r) = \frac{{e^{i\zeta _rr}}}{{\sqrt 2 }}\left( {\begin{array}{*{20}{c}} {1 + i\sin 2\varphi } & { - i\cos 2\varphi } \\ { - i\cos 2\varphi } & {1 - i\sin 2\varphi } \end{array}} \right)$$, which again exhibits an *inhomogeneous full-matrix* form. To realize such a metasurface, we follow the general design strategy discussed in Sec. 2.2 to obtain the required $\{ \xi (\vec r),{\Phi}_{{\mathrm{res}}}(\vec r),{\Delta}{\Phi}(\vec r)\}$ distributions for the metasurface,

12 $$\xi (\vec r) = \varphi + \frac{\pi }{4},{\mathrm{ }}{\Phi}_{{\mathrm{res}}}(\vec r) = \zeta _rr - \frac{\pi }{4},{\mathrm{ }}{\Delta}{\Phi}(\vec r) \equiv \frac{\pi }{2}$$

Equation (12) shows that all meta-atoms to construct such a meta-device should function as quarter-wave plates (${\Delta}{\Phi}(\vec r) \equiv \pi /2$) but exhibit different resonance phases ${\Phi}_{{\mathrm{res}}}$ and rotation angles $\xi$. Figure 7b depicts how ${\Phi}_{{\mathrm{res}}}$ and $\xi$ vary against $\vec r$ for the meta-device, which can help us determine the geometrical parameters ${\mathrm{\{ }}L_u,L_v{\mathrm{,}}\xi {\mathrm{\} }}$ of all needed meta-atoms based on a phase diagram similar to Fig. 4d but for $\lambda = 1064\;{\mathrm{ nm}}$. With all meta-atoms determined (see all geometrical parameters in Table S5 in the Supplementary Information), we finally obtain the meta-device design.

We now experimentally verify our theoretical predictions using the setup schematically depicted in Fig. 7c. As the meta-device can convert normally incident LCP light into a “driven” SW bound at the metasurface, we need to use a plasmonic metal to efficiently guide the generated SW out since otherwise, the SW will be bounced back and scattered to the far field at the device edge, making experimental characterizations difficult to carry out. The guiding-out plasmonic metal is the same Au layer as in our MIM structures but covered with a dielectric (SiO_2_) layer of thickness $h_d = 100\;{\mathrm{nm}}$. We found that such a plasmonic metal supports a branch of surface plasmon polaritons (SPPs) with parallel wavevectors well matching the designed *radial phase gradient* $\zeta _r$ of our meta-device at $\lambda = 1064\,{\mathrm{nm}}$ (see upper right inset in Fig. 7c), yielding the best guiding-out performance. Therefore, the finally fabricated sample contains two parts—the central part of radius $2.04\lambda$ occupied by the meta-device and the remaining part occupied by the above-designed plasmonic metal (see upper left inset in Fig. 7c for an SEM image of the sample).

We first employ homemade leakage radiation microscopy (LRM) to map out the generated SW and SPP field patterns in our system (see Fig. 7c) when the meta-device is illuminated by a normally incident LCP Gaussian beam with a spot size *w*_0_ = 4.9 ± 0.4 μm. The divergence of the incident light in near-field experiments is less than 10 degrees. Figure 7d depicts the image recorded by our LRM, clearly showing that strongly driven SWs are generated on the metasurface (area inside the dashed circle), which are then guided out to flow as eigen-SPPs on the plasmonic metal (area outside the dashed circle). To experimentally estimate the propagation length of the generated SPPs, we first evaluate the average SPP intensities $\bar I_r$ on circles with different radii r (Fig. 7d) and then depict in Fig. 7e how the obtained $\bar I_rr$ varies against $r$. Fitting the $\bar I_rr\sim r$ curve with $\ln (\bar I_rr) = \ln (S) - r/L_p$, where S is an intensity constant (see Fig. 7e), we can readily obtain the SPP propagation length $L_p$, which is 22–28 μm. Next, using the method presented in^32^, we find that the working efficiency of our SPP coupler is 34 ± 6% (see Sec. 7.2 in the SI for detailed analyses). The numerical simulations are in reasonable agreement with the experimental results and further demonstrate that the meta-coupler works in a broad wavelength band (930–1400 nm), exhibiting a maximum efficiency of 61.4% at the target wavelength of 1064 nm (see Sec. 7.3 in the SI). The discrepancy between experiments and simulations might relate to fabrication imperfections and nonideal plane wave input.

**Fig. 8:**
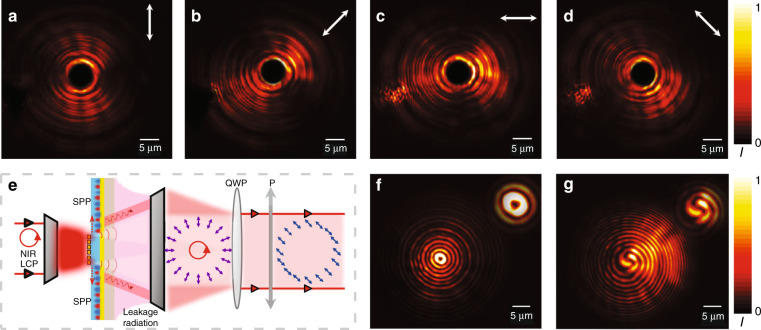
Characterization of the vectorial and vortex properties of the generated VOF. Optical images recorded by our LRM for the generated VOF after passing through a linear polarizer with a tilt angle of **a** 0°, **b** 45°, **c** 90° and **d** 135°. **e** Schematic of the setup for the interference experiment. **f** Optical image recorded by our LRM for the generated VOF after the polarization-filter procedure, as schematically shown in **e**. The donut shape with an intensity zero in the middle is a typical sign of a vortex beam. **g** Interference pattern between transmitted light and a spherical wave, recorded by our LRM. The insets in **f** and **g** depict zoomed-in pictures of the recorded patterns

We continue to experimentally characterize the *polarization distribution* of the generated near-field VOF by adding a rotatable linear polarizer in front of our CCD. As shown in Fig. 8a–d, the measured polarizer-filtered intensity profiles are consistent with our theoretical expectation that the local polarizations must be parallel to the radial direction (more experimental results can be found in Sec. 7.4 of the Supplementary Information).

We finally experimentally characterize the *vortex properties* of the generated near-field VOF. The working principle of the LRM technique naturally requires that our meta-device cannot be totally reflective, so the light signal received by our CCD contains not only the desired SW generated on the meta-device but also the directly transmitted LCP light. However, the latter does not exhibit the expected OAM property. To filter this LCP light component out, we add a quarter-wave plate to first change the light polarization from LCP to LP and then use another linear polarizer to project this LP component out (see Fig. 8e). However, such a procedure inevitably filters out *all* LCP components inside the received light signals (including the directly transmitted light and the generated SW), so the VOF light signal now received exhibits homogeneous LP rather than cylindrical polarization, as schematically shown in Fig. 8e. Figure 8f shows the CCD-captured LRM image of our VOF after undergoing the LCP-filtering procedures. The recorded pattern exhibits a well-defined doughnut shape with a minimum at the center, well illustrating the vortex nature of the generated SW. We finally characterize the orbital momentum of the generated SW by performing an interference measurement with a homemade Michelson interferometer (see Sec. 8 in the SI for the experimental setup). The interference between the LCP-filtered VOF and a spherical wave generates a pattern containing a 2^nd^-order spiral shape (see Fig. 8g), which proves that the LCP-filtered VOF exhibits OAM with $l = 2$. The interference experiment result with a quasi-plane wave generating a 2^nd^-order fork pattern can be found in Sec. 7.5 in the SI. The topological charge is not $l = 1$ as expected simply because here, we have filtered out the LCP component from the generated VOF, which does not exhibit any OAM. Recombining the LCP component (carrying no OAM) and RCP component (carrying OAM with $l = 2$) with the same amplitudes, we can thus construct the whole VOF exhibiting OAM with $l = 1$ (see Sec. 7.6 of the Supplementary Information for detailed analyses).

## Discussions

We mention several important points before closing this section. First, we note that the strategy proposed in Sec. 2.1 is so generic that we can design VOF-generation meta-devices working for impinging light with *arbitrary* incident angles and polarizations. In fact, the meta-devices realized in Sec. 2.3 and Sec. 2.4 are for incident light with LP and LCP, respectively. Meanwhile, we can not only practically realize the model meta-device proposed in Sec. 2.1 working for LCP incidence but also further design a meta-device achieving the same functionality as in Sec. 2.1 but working for incident light with an elliptical polarization (see Sec. 9 in the SI for the designs of two meta-devices). Furthermore, such meta-devices can generate a VOF with an inhomogeneous distribution of *elliptical* polarizations, well complimenting the effects experimentally demonstrated in Sec. 2.3 and 2.4. Second, we note that while the design strategy proposed in Sec. 2.1 is established for ideal lossless cases, our practically fabricated meta-devices, formed by realistic metals, still work well in creating the designed VOFs, although with nonideal working efficiencies. To enhance the working efficiencies of the devices, low-loss metals or dielectrics are needed to construct the meta-atoms.

In summary, by exploiting the full degrees of freedoms provided by the full-matrix inhomogeneous Jones matrix, we establish a *general strategy* to realize meta-devices to generate *VOFs* both in the *near-* and *far-fields*, with any designed *wavefronts* and *local polarization distributions*. After illustrating our generic concept by both model-level analytical calculations and benchmark experiments on an anomalously reflecting half-wave plate, we demonstrate the full capabilities of our approach by experimentally realizing two meta-devices. The first device can generate a complex far-field VOF exhibiting a vortex wavefront and an inhomogeneous distribution of elliptical polarizations, while the second can generate a special near-field VOF—a cylindrically polarized vortex SPP. All features of the generated VOFs are experimentally demonstrated, illustrating the good performance, broad bandwidth and versatile functionalities of the fabricated devices. Our results offer a systematic approach to design ultracompact optical devices to generate arbitrary VOFs under general conditions in different frequency domains, which are of great importance in both fundamental research and photonic applications.

We note that the efficiencies of plasmonic meta-devices sensitively depend on the ohmic losses of metals at different frequencies, and meta-devices might become less efficient at optical frequencies. However, one can always solve the issue by using dielectric meta-atoms to construct meta-devices following the general strategy developed here. A drawback of our approach is that it is not able to control the local amplitude of reflected light. Adding a control ability for the local amplitude can surely offer the designed metasurfaces stronger capabilities in generating arbitrary VOFs with better properties, which is a very interesting future project. Many future works can be expected following our work, such as extending the concept to the transmission geometry, off-normal incidences, and arbitrary incident polarizations and applying the generated VOFs to multichannel communications, near-field sensing, optical trapping, and superresolution imaging.

## Materials and methods

### Numerical simulation

We performed finite-difference time-domain simulations using numerical software. The permittivity of Au was described by the Drude model $\varepsilon _r(\omega ) = \varepsilon _\infty - \frac{{\omega _p^2}}{{\omega (\omega + i\gamma )}}$, with $\varepsilon _\infty = 9,\omega _p = 1.367 \times 10^{16}s^{ - 1},\gamma = 1.224 \times 10^{14}s^{ - 1}$, obtained by fitting to experimental results. The SiO_2_ spacer was considered a lossless dielectric with permittivity $\varepsilon = 2.085$. Additional losses caused by surface roughness and grain boundary effects in thin films as well as dielectric losses were effectively considered in the fitting parameter $\gamma$. Absorbing boundary conditions were implemented to remove the energy of those SWs flowing outside the simulation domain.

### Sample fabrication

All MIM trilayer samples were fabricated using standard thin-film deposition and electron-beam lithography (EBL) techniques. We first deposited 5 nm Cr, 125 nm Au, 5 nm Cr and an 180 nm SiO_2_ dielectric layer onto a silicon substrate using magnetron DC sputtering (Cr and Au) and RF sputtering (SiO_2_). Second, we lithographed the cross structures with EBL, employing an ~100 nm thick PMMA2 layer at an acceleration voltage of 20 keV. After development in a solution of methyl isobutyl ketone and isopropyl alcohol, a 5 nm Cr adhesion layer and a 30 nm Au layer were subsequently deposited using thermal evaporation. The Au patterns were finally formed on top of the SiO_2_ film after a lift-off process using acetone.

### Experimental setup

We used a near-infrared microimaging system to characterize the performance of all designed meta-atoms. A broadband supercontinuum laser (Fianium SC400) source and a fiber-coupled grating spectrometer (Ideaoptics NIR2500) were used in far-field measurements. A beam splitter, a linear polarizer and a CCD were also used to measure the reflectance and analyse the polarization distributions.

A homemade NIR macroscopic angular resolution spectroscope was employed for anomalous-reflection meta-wave plate characterizations. The size of the incident light spot was minimized to 130 μm. While the sample was placed on a fixed stage, the fiber-coupled receiver equipped with a polarizer was placed on a motorized rotation stage to collect the reflected signal in the right direction.

An NIR microimaging system with a homemade Michelson interferometer was employed to perform real-time imaging of the far-field VOF and its interferences with the reference light.

For the near-field characterization, a typical LRM system combined with a Michelson-type interferometer was employed for real-time imaging of the excited SPP and its interference with the reference light.

## Supplementary information

SUPPLEMENTAL MATERIAL

Movie 1
