# Supplementary material for: Efficient generation of complex vectorial optical fields with metasurfaces
Source: Light Sci Appl. 2021 Mar 31;10:67. doi: 10.1038/s41377-021-00504-x (PMC8012391; doi:10.1038/s41377-021-00504-x)
Supplement: Supplementary file 1 — SUPPLEMENTAL MATERIAL [file 41377_2021_504_MOESM1_ESM.docx]

Supplementary Information for

**Efficient Generation of Complex Vectorial Optical Fields with Metasurfaces**

Dongyi Wang^1†^, Feifei Liu^1†^, Tong Liu^1†^, Shulin Sun^2*^, Qiong He^1,3,4*^, Lei Zhou^1,3,4*^

^1^. State Key Laboratory of Surface Physics, Key Laboratory of Micro and Nano Photonic Structures (Ministry of Education) and Department of Physics, Fudan University,
Shanghai 200438, China

^2^.  Shanghai Engineering Research Centre of Ultra-Precision Optical Manufacturing, Green Photonics and Department of Optical Science and Engineering, Fudan University, Shanghai 200433, China

^3^. Academy for Engineering and Technology, Fudan University, Shanghai 200433, China

^4^. Collaborative Innovation Centre of Advanced Microstructures, Nanjing 210093, China

*Corresponding Authors: Shulin Sun, Email: sls@fudan.edu.cn

Qiong He, Email: [qionghe@fudan.edu.cn](mailto:qionghe@fudan.edu.cn)

Lei Zhou, E-mail: [phzhou@fudan.edu.cn](mailto:phzhou@fudan.edu.cn)

†These authors contributed equally to this work

Section 1. Supplementary information for Jones matrix analyses

**1.1 Derivations of Eq. (1) in the main text**

In the lossless case, the considered system exhibits time-reversal symmetry. Suppose $\hat{\text{K}}$ is the time-reversal operator, corresponding to a simple operation of taking a complex conjugation on the wave-function. We now derive the constraints imposed on the Jones matrix **R**. Consider the scattering process $|\mathrm{ref}\rangle=\text{R}|\mathrm{inc}\rangle$ where $|\mathrm{inc}\rangle$ and $|\mathrm{ref}\rangle$ represent the incident and reflected wave-functions. The time-reversal states of these two wave-functions are $|\text{ref}\rangle=\hat{\text{K}}|\text{inc}\rangle$ and $|\text{inc}\rangle=\hat{\text{K}}|\text{ref}\rangle$, respectively. Since the time-reversal states also satisfy the same scattering process, we thus get that

$\hat{\mathrm{K}}=\text{R}\hat{\mathrm{K}}\text{R}$. (S1)

Energy conservation condition can be expressed by

$\hat{\text{R}}^{\text{†}}\hat{\text{R}}=\hat{I}$ (S2)

with $\hat{\text{R}}^{\text{†}}$ being the conjugate transpose of $\hat{\text{R}}$. According to Eqs. (S1-S2), we have

$\hat{\text{R}}^{\text{†}}\hat{\mathrm{K}}=\hat{\mathrm{K}}\hat{\text{R}}$ (S3)

yielding that

$\left|r_{xy}\right|=\left|r_{yx}\right|,\Phi_{xy}=\Phi_{yx}$ (S4)

Substituting Eq. (S4) into Eq. (S2), we finally obtain

$\left\{\begin{matrix} \left|r_{xx}\right|^{2}+\left|r_{yx}\right|^{2}=1,\left|r_{xy}\right|^{2}+\left|r_{yy}\right|^{2}=1 \\ 2\Phi_{xy}=\Phi_{xx}+\Phi_{yy}+\left(2n+1\right)\pi\end{matrix}\right.$ (S5)

After combining Eqs. (S4) and (S5), we thus obtain Eq. (1) in the main text

**1.2 Derivations of Eqs. (6-7) in the main text**

According to the definition of $\tilde{\mathbf{R}}\text{(}\xi\text{,}\Phi_{\text{res}}\text{,}\Delta\Phi\text{)}$, we can explicitly derive out Eq. (6) in the main text as:

$\tilde{\mathbf{R}}(\xi,\Phi_{\text{res}},\Delta\Phi)|\sigma_{0}\rangle=e^{i\left(\Phi_{\text{res}}+\frac{\pi}{4}\right)}\left(\begin{matrix} s_{1}(\Delta\Phi,\xi;\Theta_{0},\Psi_{0}) \\ s_{2}(\Delta\Phi,\xi;\Theta_{0},\Psi_{0}) \end{matrix}\right)$ (S6)

where

$\left\{\begin{matrix} s_{1}(\Delta\Phi,\xi;\Theta_{0},\Psi_{0})=e^{-\frac{i\Psi_{0}}{2}}\cos\frac{\Delta\Phi}{2}\cos\frac{\Theta_{0}}{2}-ie^{-2i\xi}e^{+\frac{i\Psi_{0}}{2}}\sin\frac{\Delta\Phi}{2}\sin\frac{\Theta_{0}}{2} \\ s_{2}(\Delta\Phi,\xi;\Theta_{0},\Psi_{0})=e^{+\frac{i\Psi_{0}}{2}}\cos\frac{\Delta\Phi}{2}\sin\frac{\Theta_{0}}{2}-ie^{2i\xi}e^{-\frac{i\Psi_{0}}{2}}\sin\frac{\Delta\Phi}{2}\cos\frac{\Theta_{0}}{2} \end{matrix}\right.$ (S7)

We now map the right-hand side of Eq. (S6) to the form of $\exp\left(i\Phi_{\text{tar}}\right)|\sigma_{\text{tar}}\rangle$:

$e^{i\left(\Phi_{\text{res}}+\frac{\pi}{4}\right)}\left(\begin{matrix} s_{1}(\Delta\Phi,\xi;\Theta_{0},\Psi_{0}) \\ s_{2}(\Delta\Phi,\xi;\Theta_{0},\Psi_{0}) \end{matrix}\right)=\exp\left(i\Phi_{\text{tar}}\right)\left(\begin{matrix} e^{-i\frac{\Psi_{\text{tar}}}{2}}\cos\frac{\Theta_{\text{tar}}}{2} \\ e^{+i\frac{\Psi_{\text{tar}}}{2}}\sin\frac{\Theta_{\text{tar}}}{2} \end{matrix}\right)$. (S8)

After solving Eq. (S8), we get:

$\begin{matrix} \Theta_{\text{tar}}=2\cos^{-1}\left|s_{1}\right| \\ \text{For }s_{1}s_{2}=0:\text{ }\left\{\begin{matrix} \Psi_{\text{tar}}=0 \\ \Phi_{\text{tar}}=\Phi_{\text{res}}+\frac{\pi}{4}+\frac{1}{i}\log\left(s_{1}+s_{2}\right) \end{matrix}\right. \\ \text{For }s_{1}s_{2}\neq0:\text{ }\left\{\begin{matrix} \Psi_{\text{tar}}=\frac{1}{i}\log\left[\frac{s_{2}}{s_{1}}\cot\left(\cos^{-1}\left|s_{1}\right|\right)\right] \\ \Phi_{\text{tar}}=\Phi_{\text{res}}+\frac{\pi}{4}+\frac{1}{4i}\log\left(\frac{s_{1}s_{2}}{s_{1}^{*}s_{2}^{*}}\right) \end{matrix}\right. \end{matrix}$ (S9)

In the special case of the LCP light illumination (i.e., $(\Theta_{0},\Psi_{0})=(0,0)$), Eq. (S9) can be simplified to the following form:

$\left\{\begin{matrix} \Theta_{\text{tar}}=\Delta\Phi\\ \Psi_{\text{tar}}=2\xi-\frac{\pi}{2} \\ \Phi_{\text{tar}}=\Phi_{\text{res}}+\xi\end{matrix}\right.$ (S10)

for $\Delta\Phi\neq\pm\pi$. We thus obtain Eq. (6) and Eq. (7) in the main text.

It is worth mentioning that for the LCP light illumination case but with $\Delta\Phi=\pm\pi$(indicating that the constitutive meta-atom supports perfect polarization conversion), Eq. (S10) should be modified to the following form:

$\left\{\begin{matrix} \Psi_{\text{tar}}=0 \\ \Phi_{\text{tar}}=\Phi_{\text{res}}+\frac{\pi}{4}-\frac{\Delta\Phi}{2}+2\xi=\Phi_{u}+2\xi\end{matrix}\right.$ (S10’)

One may notice the difference between Eqs. (S10) and (S10’). In the case of$\Delta\Phi\neq\pm\pi$, polarization eigenstates corresponding to different $\xi$ are $\left(\begin{matrix} e^{-i\left(\xi-\pi/4\right)}\cos(\Delta\Phi/2) \\ e^{i\left(\xi-\pi/4\right)}\sin(\Delta\Phi/2) \end{matrix}\right)$, which are non-degenerate and contain phase factors of $e^{\pm i\xi}$. In contrast, for the special case of $\Delta\Phi=\pm\pi$, all polarization eigenstates $\left(\begin{matrix} 0 \\ e^{i\left(\xi-\pi/4\right)} \end{matrix}\right)$ are degenerate and denoting the same RCP state apart from a phase factor $e^{i\xi}$. Therefore, we can extract such a phase term out and put it into the Berry phase. As a result, in such a case, $\Phi_{\text{tar}}$ contains a term $2\xi$ rather than $\xi$.

Section 2. Green’s function approach

**
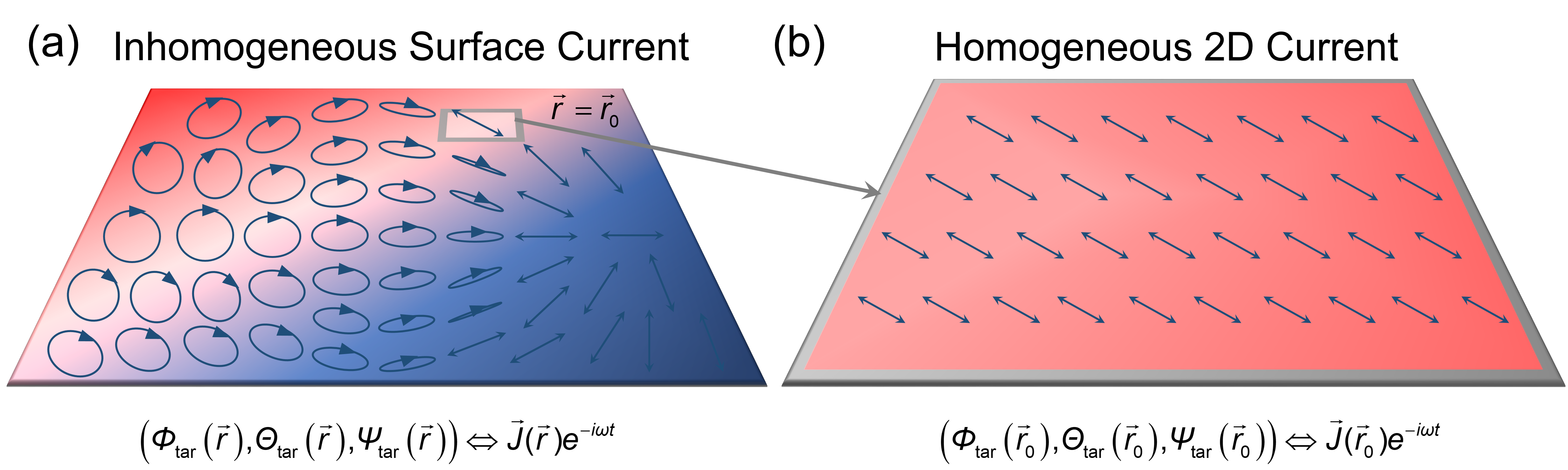
**

**Fig. S1 Schematics of the surface current analysis based on GF approach.** (a) An inhomogeneous surface current $\vec{J}(\vec{r})e^{-i\omega t}$ is induced on the model system under the illuminations of an EM wave. (b) A homogeneous 2D current $\vec{J}(\vec{r}_{0})$ yielding the radiating fields possessing phase/polarization property determined by $\left(\Phi_{\text{tar}}(\vec{r}_{0}),\Theta_{\text{tar}}(\vec{r}_{0}),\Psi_{\text{tar}}(\vec{r}_{0}) \right)$.

We describe the Green’s function (GF) approach to calculate the waves scattered by our model meta-devices. Illuminated by a normally incident EM wave of frequency$\omega$, the model system with $\left(\Phi_{\text{tar}}(\vec{r}_{0}),\Theta_{\text{tar}}(\vec{r}_{0}),\Psi_{\text{tar}}(\vec{r}_{0}) \right)$ distributions will generate certain inhomogeneous surface current $\vec{J}(\vec{r})e^{-i\omega t}$, which, in turn, generates the reflected wave (see Fig. S1a). To determine the current at a local point $\vec{r}_{0}$ (i.e. $\vec{J}(\vec{r}_{0})$), we require that an infinite sheet with homogeneous current $\vec{J}(\vec{r}_{0})$ (see Fig. S1b) yields the reflected wave exhibiting the phase/polarization property determined exactly by homogeneous distributions $\left(\Phi_{\text{tar}}(\vec{r}_{0}),\Theta_{\text{tar}}(\vec{r}_{0}),\Psi_{\text{tar}}(\vec{r}_{0}) \right)$[1]. Based on this requirement, we obtain the following analytical form of the induced surface current

$\vec{J}(\vec{r})=J_{0}\delta(z)e^{i\Phi_{\text{tar}}(\vec{r})}\left(\hat{e}_{+}e^{-i\frac{\Psi_{\text{tar}}(\vec{r})}{2}}\cos\frac{\Theta_{\text{tar}}(\vec{r})}{2}+\hat{e}_{-}e^{+i\frac{\Psi_{\text{tar}}(\vec{r})}{2}}\sin\frac{\Theta_{\text{tar}}(\vec{r})}{2}\right)$ (S11)

where $\hat{e}_{\pm}=(\hat{x}\pm i\hat{y})/\sqrt{2}$ and $J_{0}$ is a normalization constant determined by the strength of the local incident and reflected fields. Since the model system is perfectly reflective at every local point, $J_{0}$ is a constant everywhere. With the current source given, we can thus use the dyadic GF approach to rigorously compute the wave reflected by the model system [2]:

$\vec{E}(\vec{r},\omega)=i\omega\mu_{0}\int\overleftrightarrow{G}(\vec{r},\vec{r}';\omega)\cdot\vec{J}(\vec{r}')d\vec{r}'$ (S12)

where the form of GF can be found in supplementary information of Ref. [1].


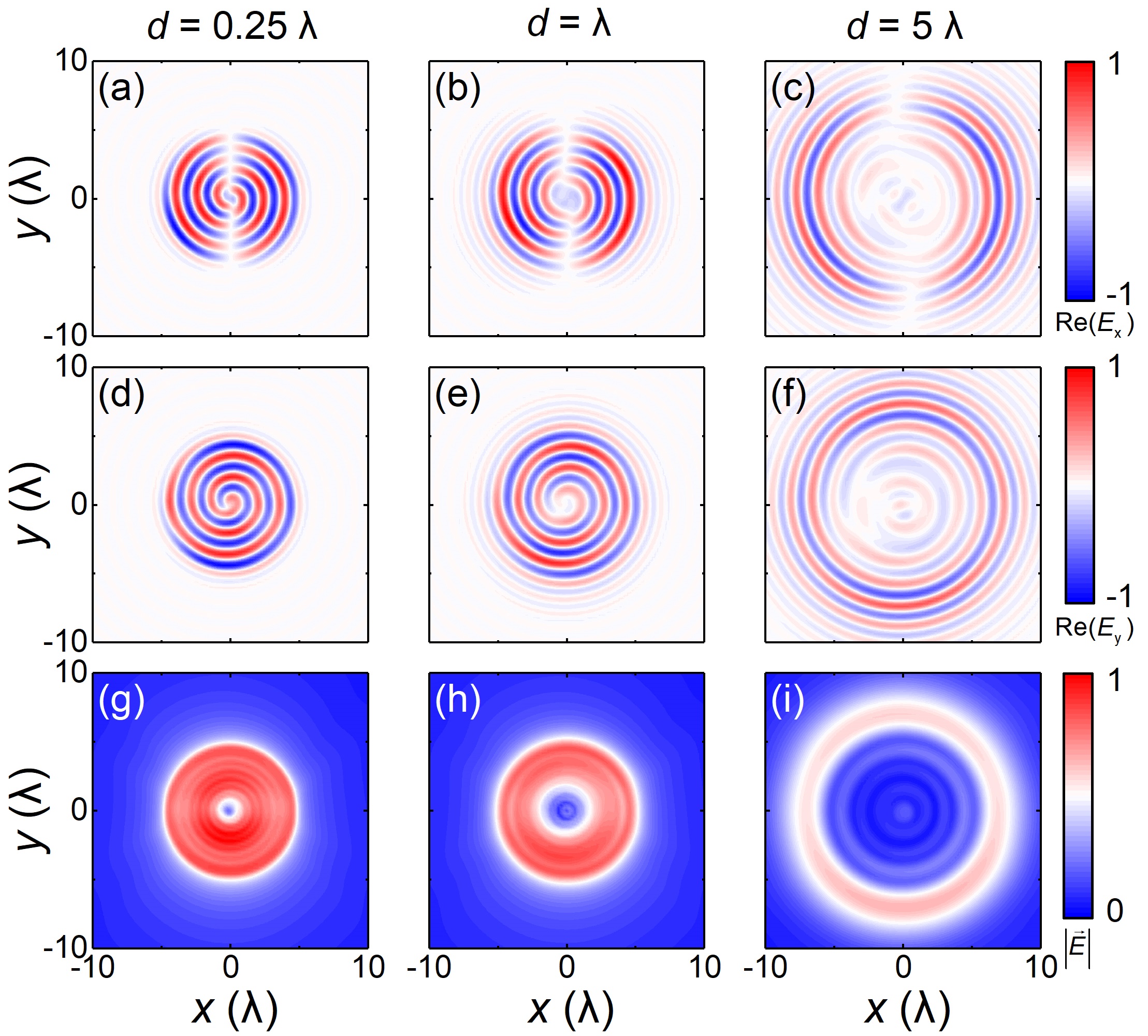


**Fig. S2 Analytical calculations on the model system with properties given by Eq. (3) in the main text under LCP incidence.** GF-calculated $\mathrm{Re}(E_{x})$ (a-c), $\mathrm{Re}(E_{y})$ (d-f) and $\text{|}\vec{E}\text{|}$ (g-i) distributions at different planes from the source with distance of $d=0.25\lambda$(a, d, g), $d=\lambda$ (b, e, h) and $d=5\lambda$(c, f, i), respectively.

We use the GF approach to calculate the field patterns on the reference planes at different distances ($d=0.25\lambda$, $d=\lambda$ and $d=5\lambda$) from the meta-device with properties given by Eq. (3), and illustrate the computed field patterns in Fig. S2.

Section 3. Detailed simulation results on MIM meta-atoms illustrated in Fig. 3


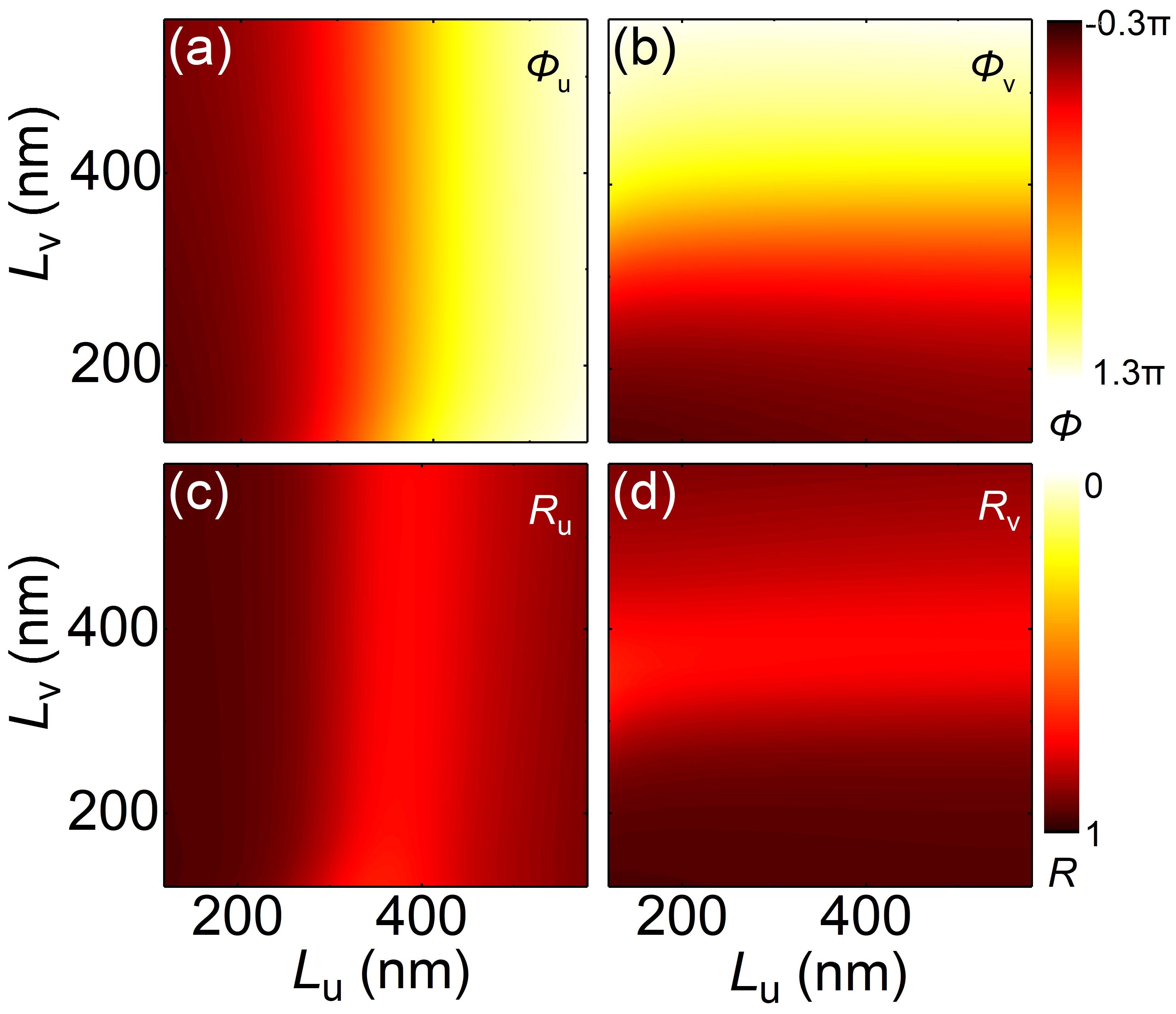


**Fig. S3** FDTD-simulated reflectance (a)$R_{u}=|r_{uu}|^{2}$ and (b)$R_{v}=|r_{vv}|^{2}$ and reflection phases (c)$\Phi_{u}$ and (d)$\Phi_{v}$ versus $L_{u}$ and $L_{v}$ for MIM meta-atoms under the illuminations of $\hat{u}$-polarized and $\hat{v}$-polarized lights respectively. The working wavelength is 1550 nm.

Section 4. Additional experimental results of the meta-atoms presented in Fig. 4

**4.1 Meta-atoms Nos. 1-4**

These meta-atoms all function as the quarter-wave plates, and their geometrical parameters are:

| Nos. | 1 | 2 | 3 | 4 |
| --- | --- | --- | --- | --- |
| *L_u_* / nm | 120 | 303 | 367 | 564 |
| *L_v_* / nm | 303 | 367 | 564 | 120 |

**Tab. S1** Geometric parameters of meta-atoms Nos. 1-4.

Based on the same technique as studying Fig. 4 in the main text, we shine the fabricated samples with LP light with its **E** field lying at an angle 45° with respect to the local u axes (i.e. $\Psi_{0}=\Theta_{0}=\pi/2$), and measured the normalized power patterns of light reflected by these meta-atoms through a rotatable linear polarizer. Measured and simulated power patterns shown in Figs. S4b, S4e, S4h and S4k reveal that the reflected lights exhibit circular polarizations in all cases, verifying that these meta-atoms all function as the quarter-wave plates with $\Delta\Phi=\pi/2$ at the wavelength of 1550 nm. In addition, both measured and simulated DOCP spectra of the reflected light illustrate that these meta-atoms all exhibit high performances within the wide working bandwidths, as shown in Figs. S4c, S4f, S4i and S4l.


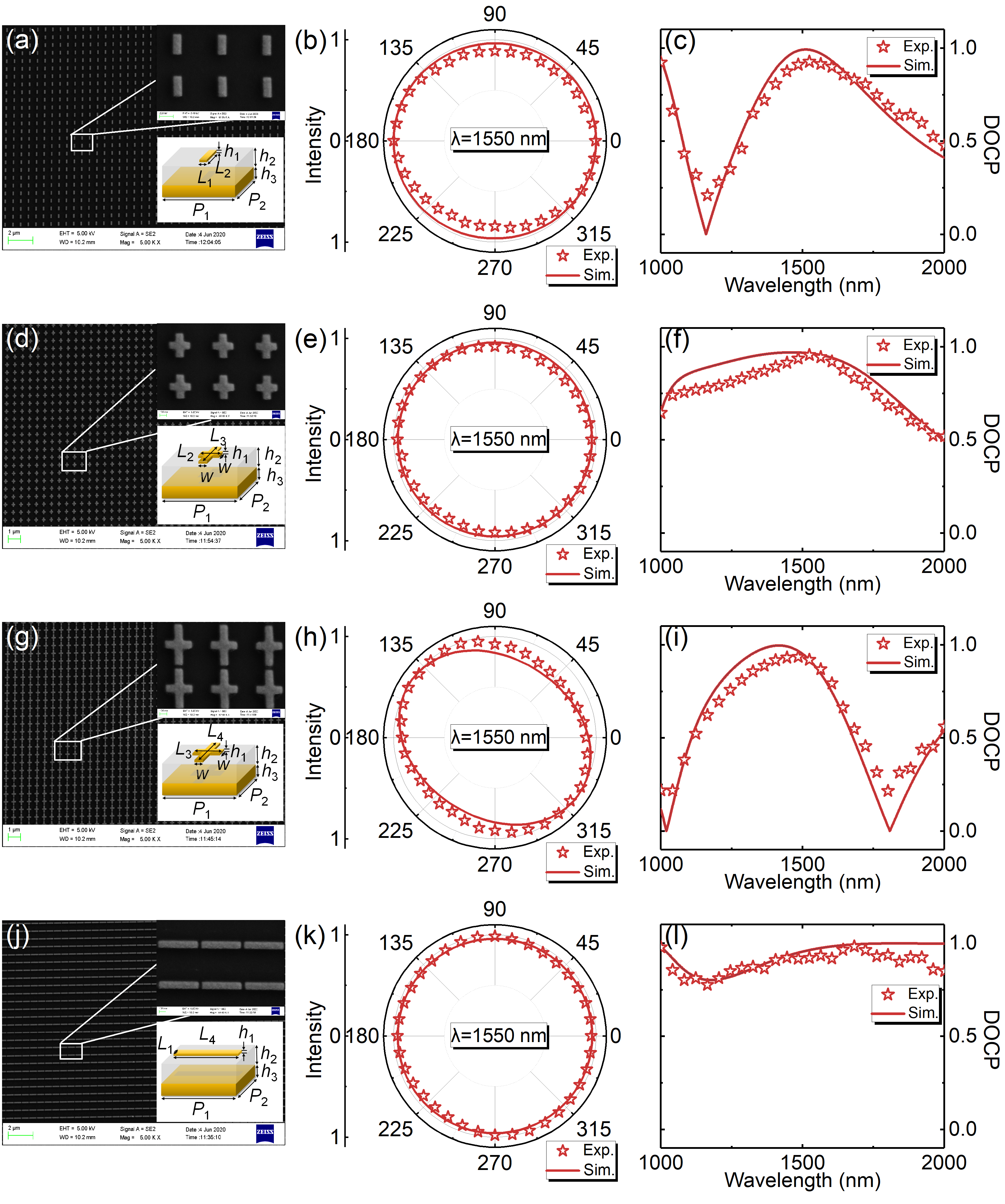


**Fig. S4** Schematics and SEM images (a, d, g, j) of four meta-quarter-wave plates formed by periodically arranged meta-atoms (Nos. 1-4) with orientation angle $\xi\text{=}0$. Normalized polarizer-filtered power patterns with the linear polarizer rotating within the range of 360° (b, e, h, k) and DOCP spectra (c, f, i, l) of the light beams reflected by four meta-quarter-wave plates, obtained by the experiments (stars) and simulations (lines) at the wavelength of 1550 nm.

**4.2 Meta-atoms Nos. 5-8**

There meta-atoms all function as the half-wave plates, and their geometric parameters are:

| Nos. | 5 | 6 | 7 | 8 |
| --- | --- | --- | --- | --- |
| *L_u_* / nm | 120 | 303 | 367 | 564 |
| *L_v_* / nm | 367 | 564 | 120 | 303 |

**Tab. S2** Geometric parameters of meta-atoms Nos. 5-8.


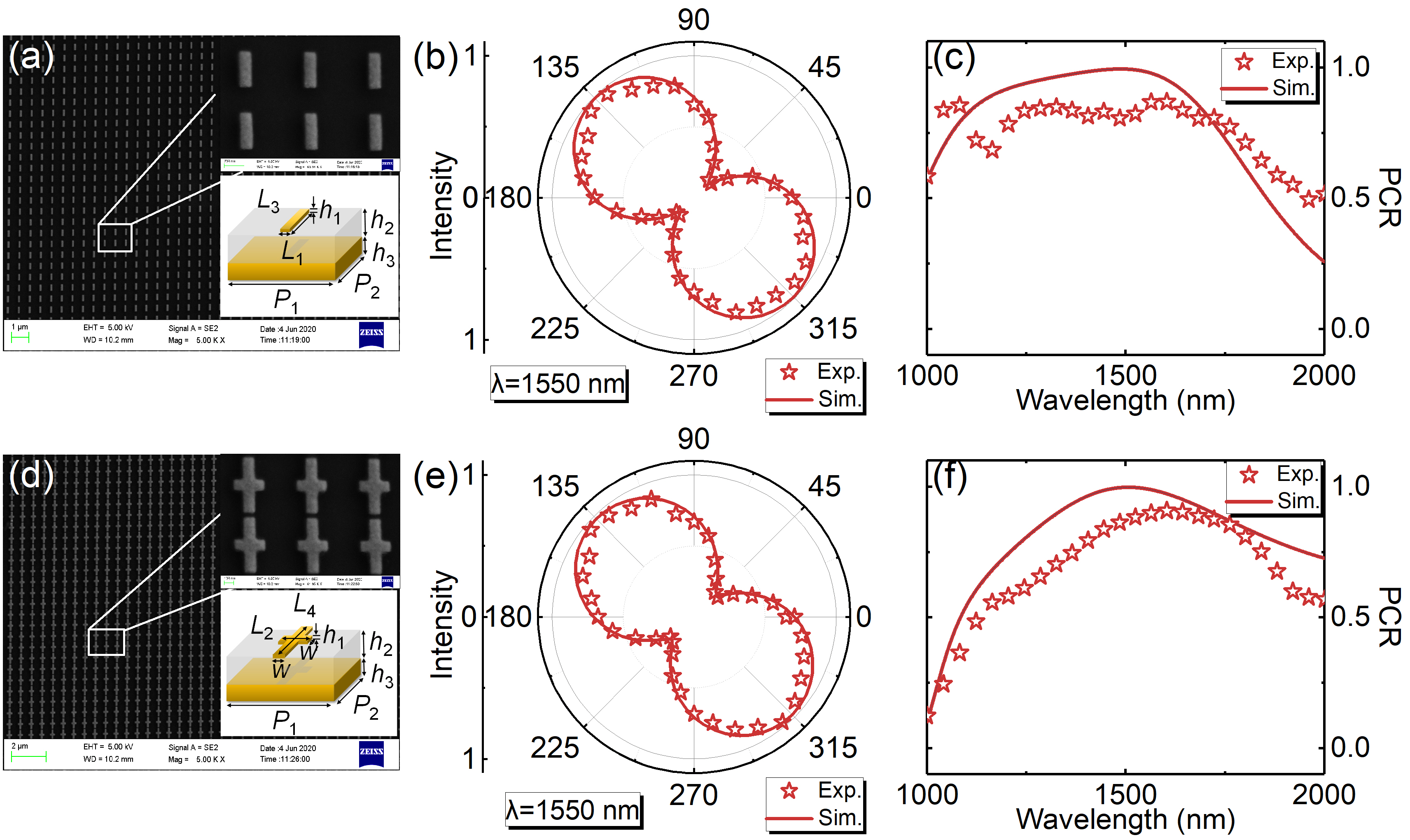


**Fig. S5** Schematics and SEM images (a, d, g, j) of two meta-half-wave plates formed by periodically arranged meta-atoms (Nos. 5, 6) with orientation angle $\xi\text{=}0$. Normalized polarizer-filtered power patterns with the linear polarizer rotating within the range of 360° (b, e) and PCR spectra (c, f) of the light beams reflected by two meta-half-wave plates, obtained by the experiments (stars) and simulations (lines) at the wavelength of 1550 nm.

We note that the meta-atoms Nos. 7 and 8 are actually identical to meta-atoms Nos. 5 and 6, only rotated by an angle of 90°. We characterized the performances of meta-atoms Nos. 5 and 6 with the same experimental technique as adopted in Sec. 4.1. Figures S5b and S5e depict the measured and simulated power patterns corresponding to these two meta-atoms with the reflected lights carrying the cross-polarized LPs, implying that these meta-atoms function as half-wave plates with $\Delta\Phi=\pi$. In addition, both measured and simulated PCR spectra indicate that these two meta-atoms exhibit high performances (PCR ~100%) within relatively broad bandwidths, as shown in Figs. S5c and S5f.

**4.3 Geometrical parameters of meta-atoms Nos. 9-12**

| Nos. | 9 | 10 | 11 | 12 |
| --- | --- | --- | --- | --- |
| *L_u_* / nm | 120 | 320 | 400 | 580 |
| *L_v_* / nm | 480 | 120 | 300 | 380 |

**Tab. S3** Geometric parameters of meta-atoms Nos. 9-12.

**4.4 Phase parameters of meta-atoms Nos. 1-12**

| Nos. | 1 | 2 | 3 | 4 | 5 | 6 | 7 | 8 | 9 | 10 | 11 | 12 |
| --- | --- | --- | --- | --- | --- | --- | --- | --- | --- | --- | --- | --- |
| $\Phi_{\mathrm{res}}/\pi$ | -0.22 | 0.13 | 0.66 | 0.31 | 0.03 | 0.46 | 0.03 | 0.46 | 0.25 | -0.16 | 0.23 | 0.71 |
| $\Delta\Phi/\pi$ | 0.42 | 0.51 | 0.54 | -1.45 | 0.81 | 0.84 | -1.19 | -1.16 | 1.33 | -0.58 | -0.64 | -0.60 |

**Tab. S4** Phase parameters of meta-atoms Nos. 1-12.

Section 5. Supplementary information for the meta-device studied in Fig. 5

**5.1 Schematics of anomalous-reflection meta-wave plate**

**
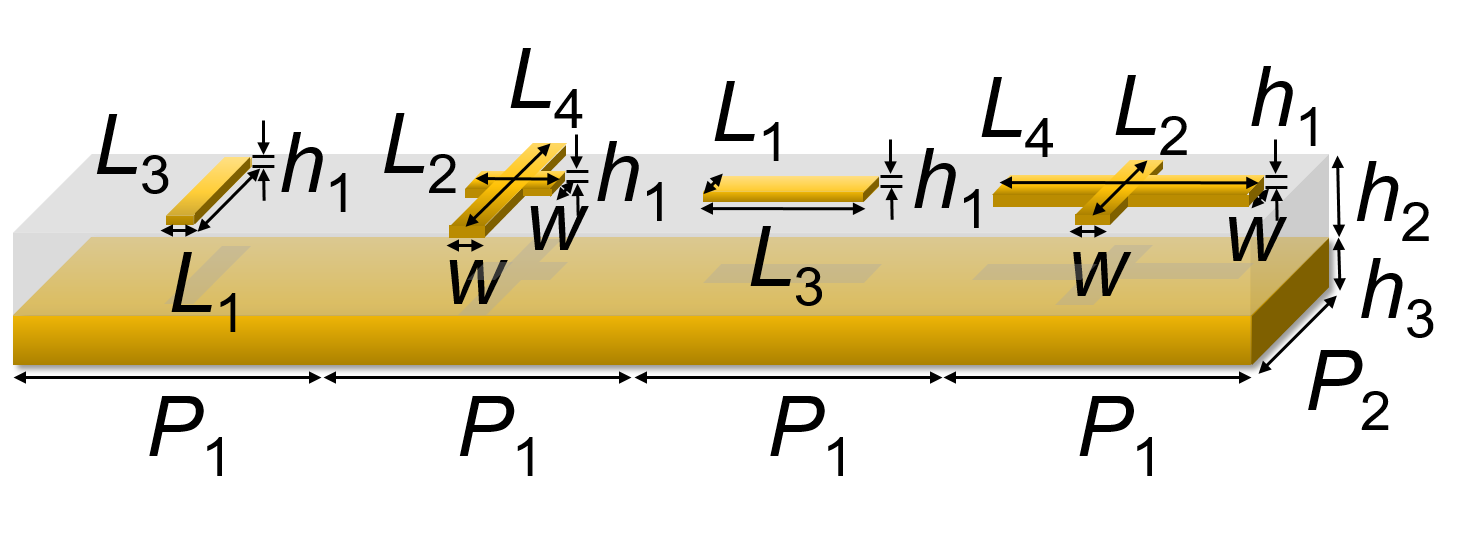
**

**Fig. S6** Schematics of the anomalous-reflection meta-wave plate studied in Fig. 5. Geometric parameters: $h_{1}=30\text{nm}$, $h_{2}=180\text{nm}$, $h_{3}=125\text{nm}$, $w=120\text{nm}$, $L_{1}=120\text{nm}$, $L_{2}=303\text{nm}$, $L_{3}=367\text{nm}$, $L_{4}=564\text{nm}$ and $P_{1}=P_{2}=600\text{nm}$.

**5.2 Simulated scattering-field distribution of the anomalous-reflection meta-wave plate**

**
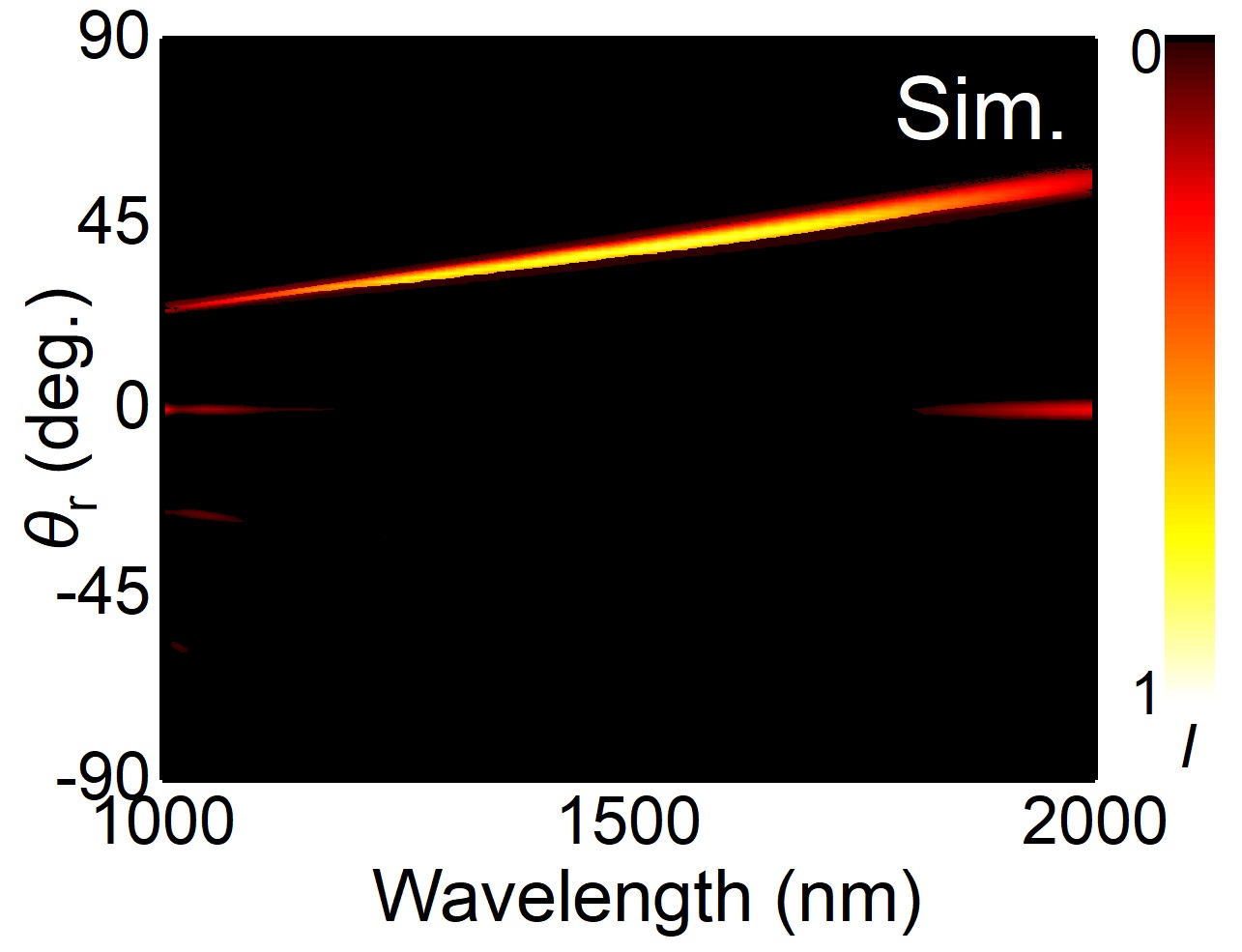
**

**Fig. S7** Simulated normalized far-field intensity versus receiving angle and wavelength of light for the metasurface depicted in Fig. 5, under the illumination of a normally incident LP light with the E-field polarized along an angle of 45° respect to the *x* axis.

Section 6. Supplementary information for the meta-device studied in Fig. 6

**6.1 Geometrical parameters**


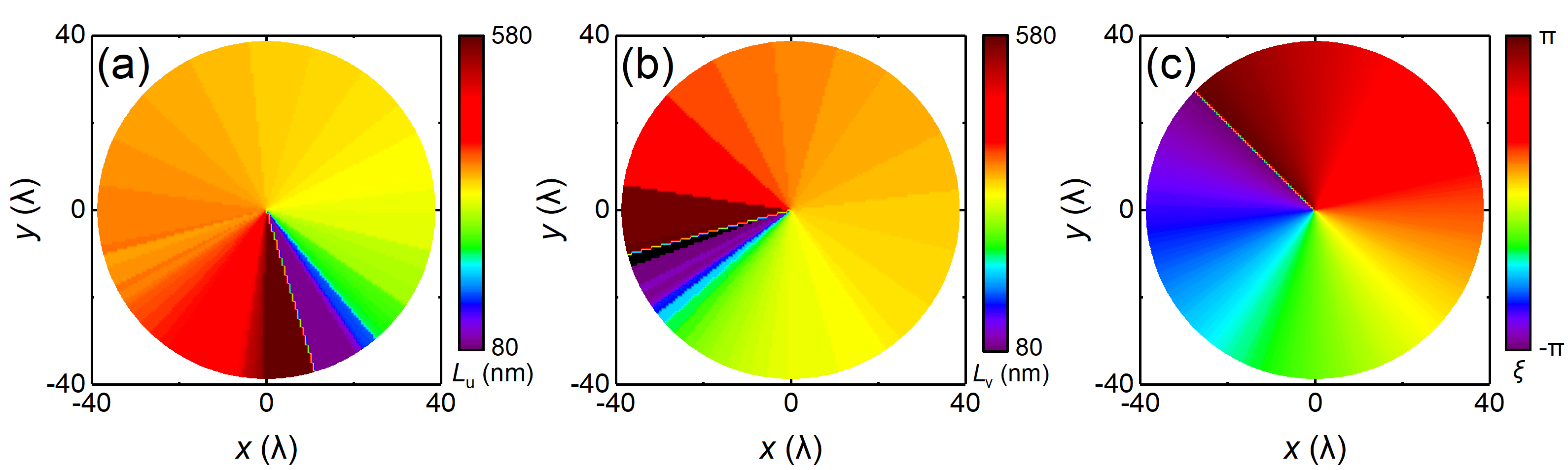


**Fig. S8** Distributions of structural details (a) $L_{u}$, (b) $L_{v}$ and (c) $\xi$ of the meta-device studied in Fig. 6 of the main text, calculated with Eq. (9) in the main text.

**6.2 More experimental results on the polarization distribution**

To illustrate clearly the properties of the polarization distribution of the generated VOF, we placed a polarizer in front of the CCD in our experiments, functioning as an analyser. As shown in the attached movie M1, as we rotate the analyser to angles varying from 0° to 360° with a step of 30°, we find that the recorded field pattern changes gradually. Specifically, the obtained patterns do not exhibit any intensity zeros, except the four particular zeros shown in Fig. 6d as the polarizer is along the horizontal direction. This fact directly demonstrates that local polarization on the wavefront of the generated VOF is an elliptical one at a general azimuthal angle, but becomes linear ones at those four particular angles, which is consistent with theoretical expectations.

Section 7. Additional information for the meta-device studied in Figs. 7-8 in the main text

**7.1 Designing meta-atoms working at 1064nm**


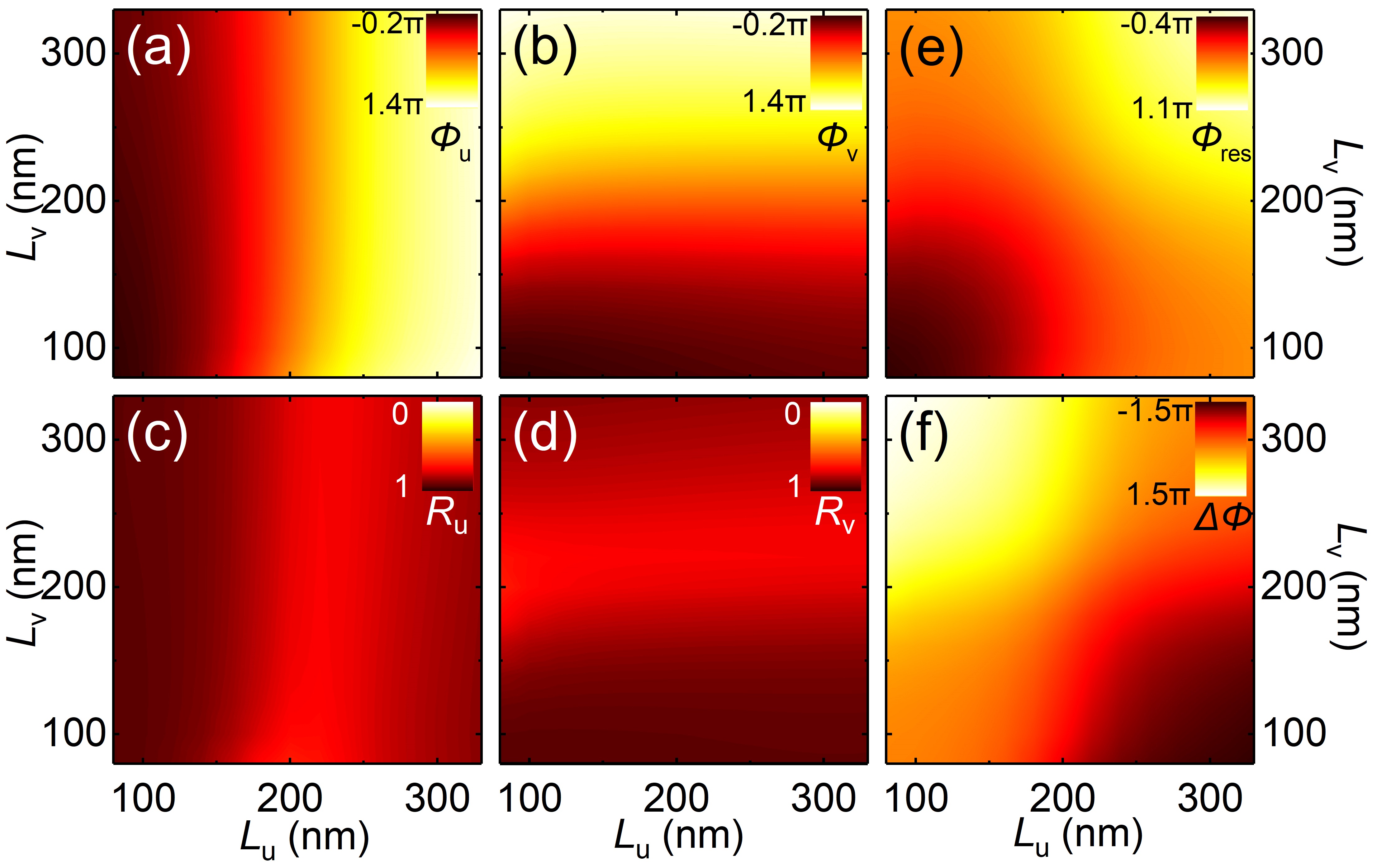


**Fig. S9** Phase diagrams of (a) $\Phi_{u}$, (b) $\Phi_{v}$, (c) $\Phi_{\text{res}}$, (d) $\Delta\Phi$, (e) $R_{u}$ and (f) $R_{v}$ versus $L_{u}$ and $L_{v}$ for the MIM meta-atoms employed in Figs 7-8, obtained by FDTD simulations at the wavelength of 1064nm.

Geometric parameters of all meta-atoms for this meta-device are listed as follows:

| Meta-atom | A | B | C |
| --- | --- | --- | --- |
| *L_u_* / nm | 156 | 220 | 334 |
| *L_v_* / nm | 204 | 294 | 80 |

**Tab. S5** Geometric parameters of meta-atoms with the bar thickness being 30nm and the bar width being 80nm.

**7.2 Experimental evaluation of surface plasmon polariton (SPP) coupling efficiency**

To quantitatively obtain the SPP coupling efficiency, we follow the approach described in Ref. [3] to carefully analyse the whole optical process in our leakage radiation microscopy (LRM) system. In our experiment, the incident beam is a collimated optical beam with Gaussian intensity distribution exhibiting a left-handed circular polarization. Such a circularly polarized beam is then focused onto the meta-coupler by a high-NA 50× Object Lens, to excite the SPP. Since the Au film at the bottom of our meta-device is purposely designed to be not too thick (i.e., 65nm), the generated SPP beam can couple to the backside of the sample, which is then decoupled to the far-field by an oil-immersion objective lens, finally being focused into the CCD by another lens.

With this LRM system, we can collect the near-field information to evaluate the propagation length *L_p_* and the excitation efficiency *C* of the generated SPP. Considering that the generated SPP is a cylindrical wave, we perform the intensity integration to obtain the average intensity $\bar{I}_{r}(r)$ collected by our CCD on a circle of radius $r$. Owing to the intrinsic loss and the decoupling effect, the total power carried by the generated SPP wave must decay exponentially as a function of *r* satisfying $\bar{I}_{r}2\pi r=S\exp(-r/L_{p})$, where *S* is a constant, *L_p_* is the propagating length of SPP, and $2\pi r$ is proportional to the wavefront size of the cylindrical SPP. The propagation length *L_p_* can be obtained by a linear fit to $\ln\left(\bar{I}_{r}2\pi r\right)$, as shown in Figure 7e in the main text. Then we can calculate the leakage radiation power of the SPP using

$P_{\text{LR}}=\int_{0}^{2\pi} \int_{0}^{\text{+}\infty} \bar{I}_{r}(r)rdrd\theta=2\pi\int_{0}^{\text{+}\infty} \frac{S\exp(-r/L_{p})}{r}rdr=2\pi S\int_{0}^{\text{+}\infty} \exp(-r/L_{p})dr=2\pi SL_{p}$(S13)

To evaluate the SPP excitation efficiency, we need to retrieve the power of launched SPP based on the integrated leakage radiation energy power $P_{\text{LR}}$. There exists two kind of loss for SPP in such an LRM system, i.e., absorption loss and leakage radiation loss, whose ratio can be expressed as $\Gamma=\mathrm{Im}(n_{\text{SPP}}^{(1)})/[\mathrm{Im}(n_{\text{SPP}}^{(2)})-\mathrm{Im}(n_{\text{SPP}}^{(1)})]$, where $n_{\text{SPP}}^{(1)}$and $n_{\text{SPP}}^{(2)}$ are the effective mode indexes of the SPP without and with leakage radiation losses, respectively. These two values can be obtained by studying two SPP meta-couplers, with corresponding bottom Au films possessing infinite and finite thicknesses, respectively. For our case (a 65-nm-thick gold film with a 100-nm glass layer at λ = 1064 nm), the loss ratio is evaluated as Γ≈13. Thus, the total power carried by the generated SPP could be retrieved by the following equation $P_{\text{SPP}}=P_{\text{LR}}(1+\Gamma)$.

We now evaluate the incident power, employing the same LRM system for fair comparison. Shine the same input Gaussian beam on a 65-nm-thick gold film, we experimentally measured the power $P_{T}$ transmitted through the gold film. Then, we evaluated the transmission rate $T$of the gold film, through comparing two integration times obtained for the CCD to receive identical signals as the gold film is removed or placed in front of the CCD, respectively. With both $P_{T}$ and $T$experimentally obtained, we then determined the total incident power via $P_{\text{in}}=P_{T}/T$. Finally, we can evaluate the SPP excitation efficiency using

$C=\frac{P_{\text{SPP}}}{P_{\text{in}}}=\frac{T(1+\Gamma)P_{\text{LR}}}{P_{T}}=\frac{2T(1+\Gamma)SL_{p}}{Iw_{0}^{2}}$ (S14)

In our case, the excitation efficiency is evaluated to be $C=34\pm6\%$.

**7.3 Simulation results for the meta-device depicted in Figs. 7 and 8**

Utilizing above-mentioned meta-atoms, we designed and fabricated the meta-device for generating vectorial vortex surface plasmon polariton (SPP) (see inset to Fig. 7c in the main text), with the simulated performances of this device shown in Fig. S10. From Fig. S10b, we can see that the incident propagating wave is converted into a vectorial vortex surface wave which is then guided out as an SPP. As shown in Figs. S10a and S10c, we find that the device functions well in a rather broad wavelength band. We also employed numerical simulations to calculate the working efficiency of our meta-device, which is defined as the ratio between the total power carried by the generated SPP and that carried by the incident light striking the meta-device. Both values are obtained by numerical integrations of power flows on appropriately designed reference planes. Numerical simulations reveal that our meta-device exhibits a maximum efficiency 61.4% at 1064 nm, and exhibits a wide working bandwidth of 930-1400nm as demonstrated in Fig. S10d.


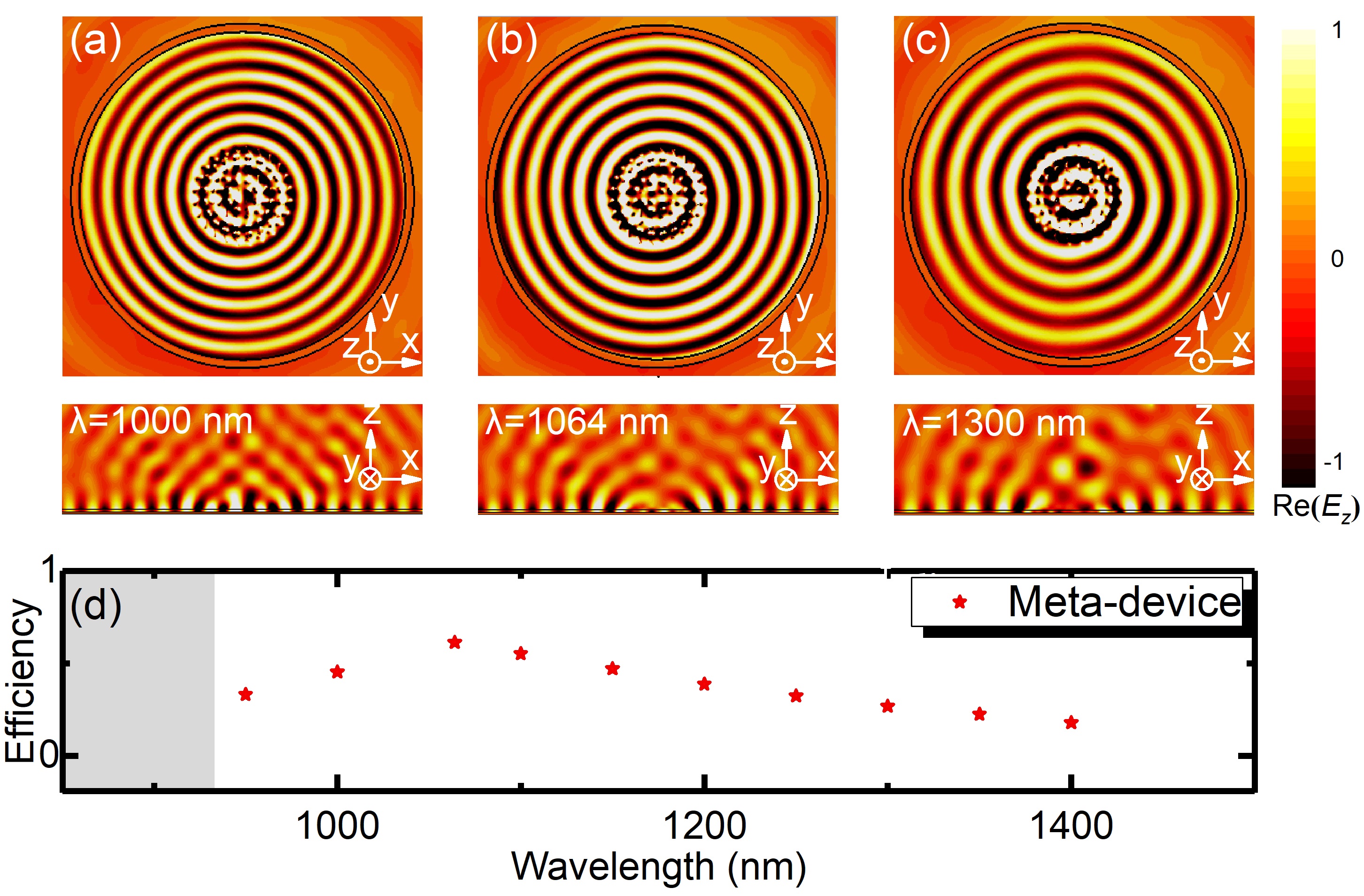


**Fig. S10** (a-c) Calculated $\mathrm{Re}\left(E_{z}\right)$ field patterns of our meta-device under the illumination of LCP light at (a) *λ*=1000nm, (b) *λ*=1064nm, (c) *λ*=1300nm, respectively. (d) Working efficiencies of our meta-device versus wavelength, computed by FDTD simulations.

**7.4 Vectorial properties of the generated VOF**

**
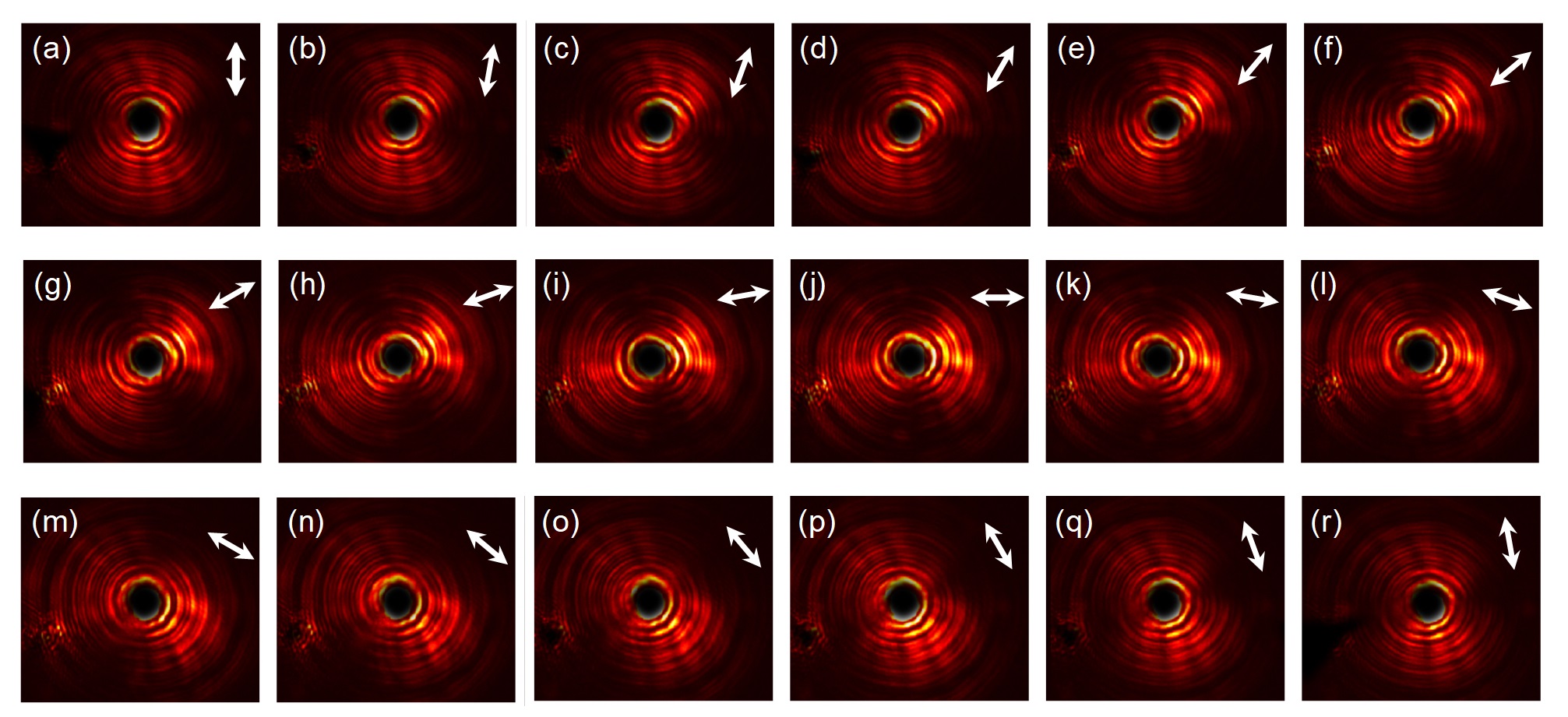
**

**Fig. S11** Recorded leakage radiation microscopy (LRM) images of the VOF generated by the meta-coupler with a linear polarizer placed in front of CCD with the tilted angle of (a) 0°, (b) 10°, (c) 20°, (d) 30°, (e) 40°, (f) 50°, (g) 60°, (h) 70°, (i) 80°, (j) 90°, (k) 100°, (l) 110°, (m) 120°, (n) 130°, (o) 140°, (p) 150°, (q) 160°, and (r) 170°, respectively.

**7.5 Interference experiment result with a quasi-plane wave**

**
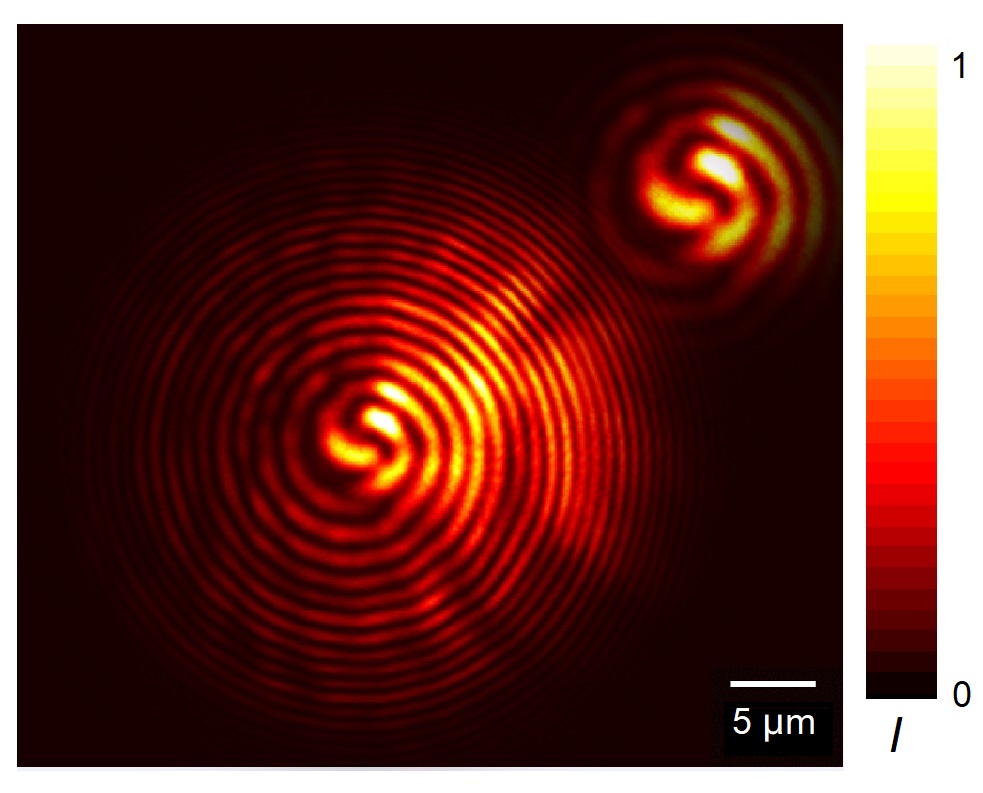
**

**Fig. S12** Measured interference pattern between the transmitted light and a quasi-plane wave, recorded by our LRM. Inset depicts the zoom-in picture of the recorded pattern.

**7.6 Detailed analyses on the OAM properties of the generated VOF**

As schematically depicted in Fig. 8e of the main text, we need to filter out the direct-transmission light in our experiment. However, this procedure also filters out the LCP component of the generated VOF, leaving only the RCP component. To identify the OAM properties of the LCP/RCP components of the generated VOF, we consider a generic meta-atom (see Fig. S13) shined by an LCP incident light.

**
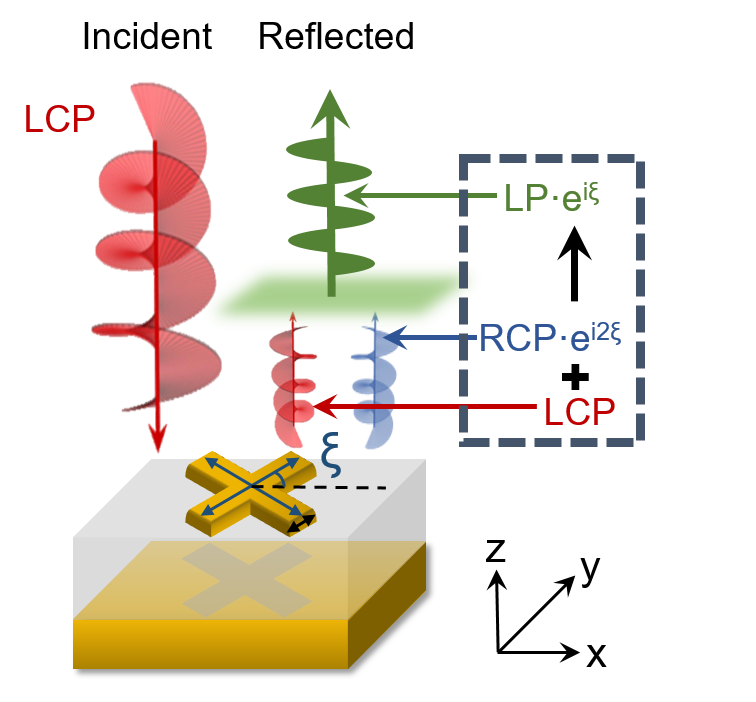
**

**Fig. S13** Schematics of the polarization conversion process and PB phases carried by the CP components with the opposite helicity.

According to the design strategy, such a meta-atom should function as a perfect quarter-wave plate and can also provide an initial phase of $\Phi_{\text{res}}+\xi$. Therefore, under the illumination of LCP light, reflected light must be linearly polarized carrying a phase of $\Phi_{\text{res}}+\xi$. To clarify the underlying physics, we can decompose such a light into LCP and RCP components as

$\vec{E}_{\text{ref}}=\frac{1}{\sqrt{2}}e^{i\left(\Phi_{\text{res}}+\frac{\pi}{4}\right)}\hat{e}_{+}+\frac{1}{\sqrt{2}}e^{i\left(\Phi_{\text{res}}+2\xi-\frac{\pi}{4}\right)}\hat{e}_{-}$ (S15)

where $\hat{e}_{\pm}$ denotes the polarization eigenstates of LCP and RCP. Interestingly, it is noted that the LCP component carries no $\xi$-dependent Berry phase and the RCP component takes a Berry phase of $2\xi$.

Considering the relation of given in Eq. (10) in the main text, we immediately draw a conclusion that the LCP component inside our generated VOF does not carry any OAM, but the RCP component carries an OAM with a topological charge of $l=2$. This explains why the experimentally measured interference pattern indicates a second-order fork, which is consistent with the OAM property of the RCP component of our generated VOF.

Section 8. Schematics of our experimental setups

**
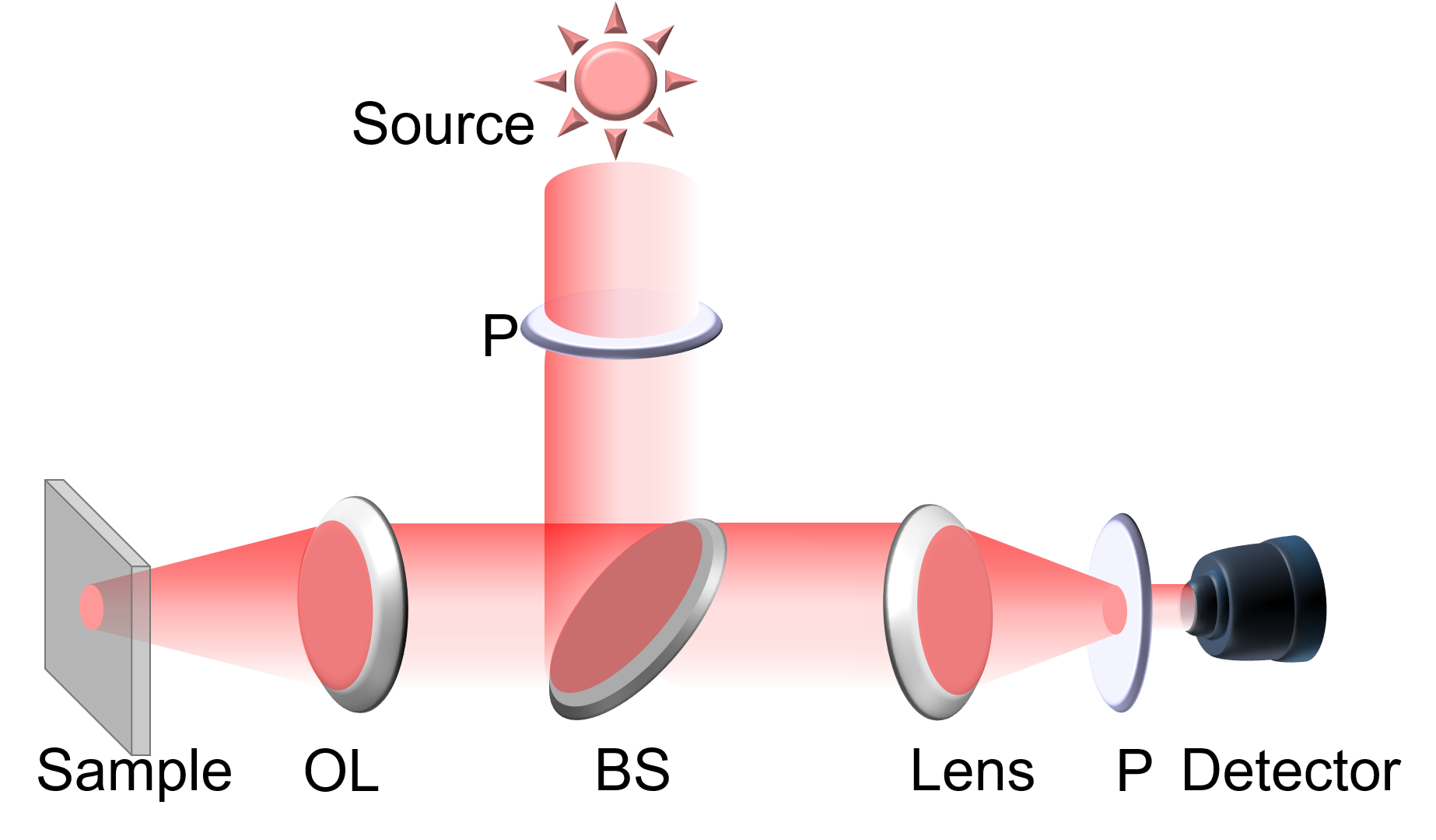
**

**Fig. S14** Schematics of the experimental setup for meta-wave plate characterization. P represents Polarizer, OL represents Objective Lens, and BS represents Beam Splitting lens.


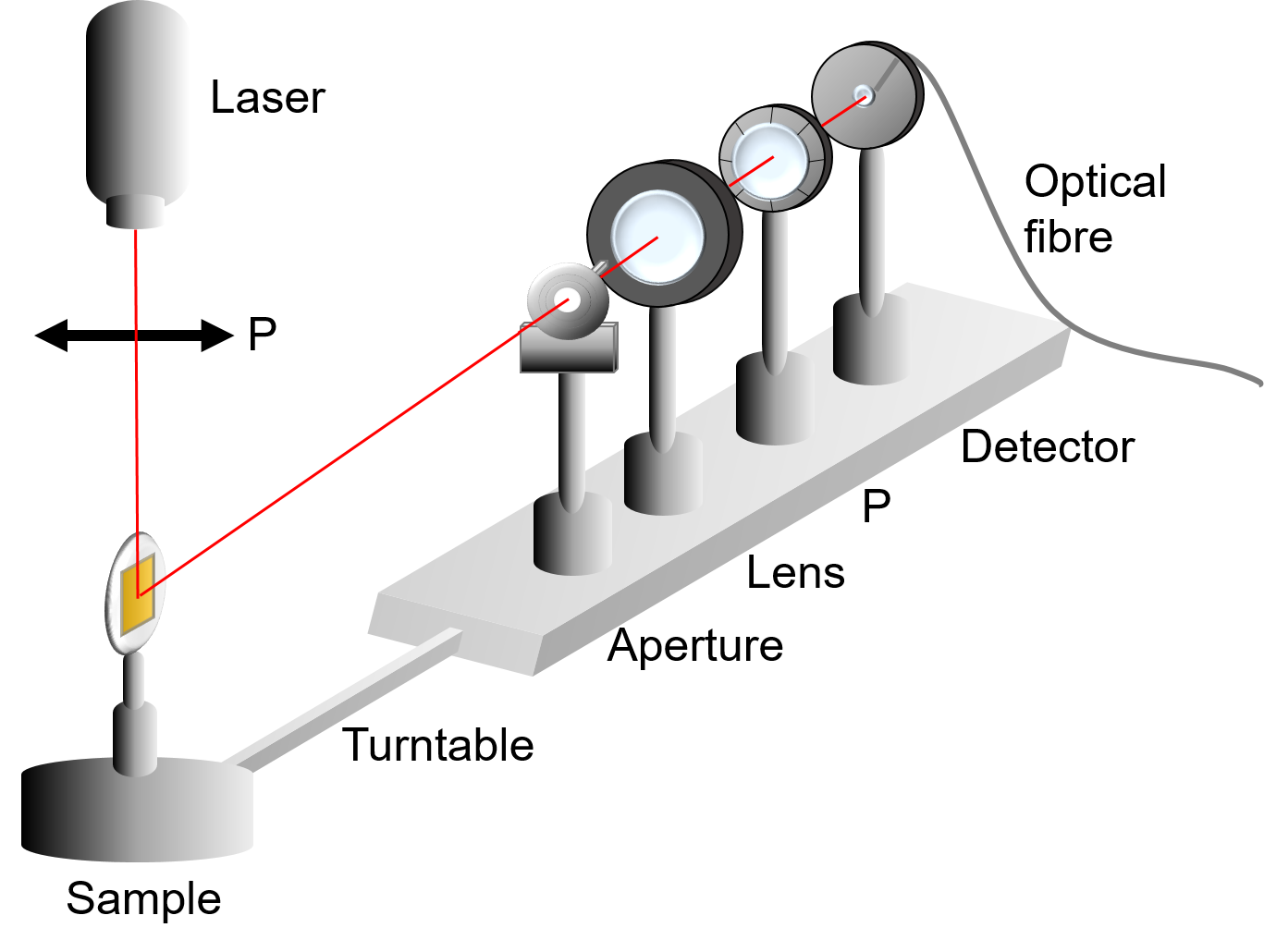


**Fig. S15** Schematic of the macro-angular-resolution spectroscopy. P represents polarizer.


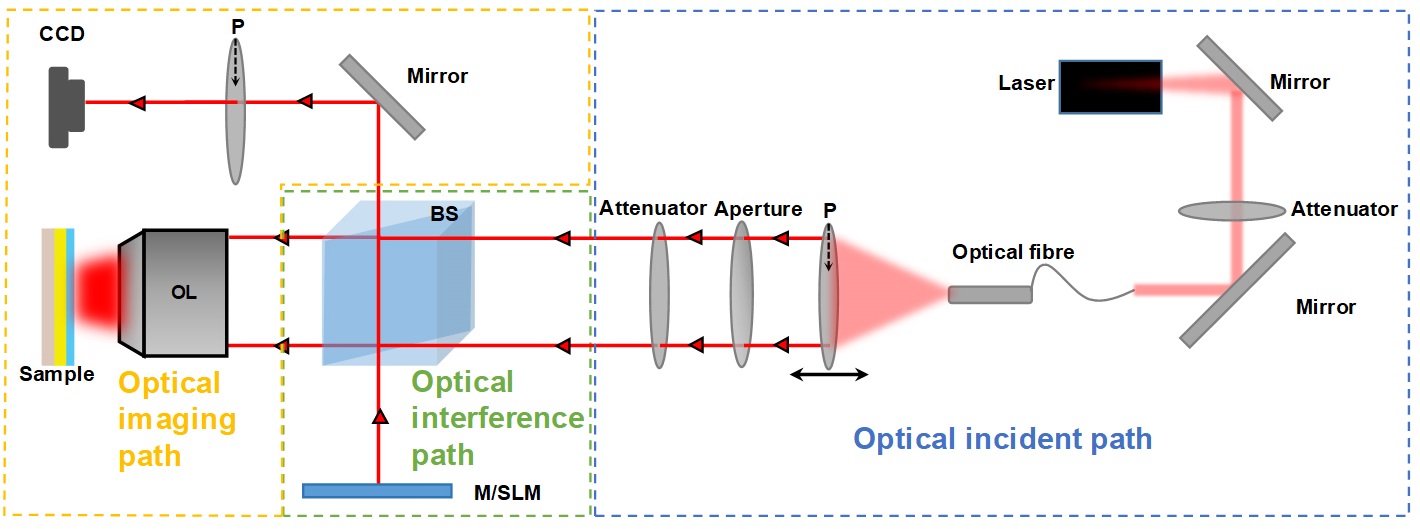


**Fig. S16** The micro-imaging system with a home-made Michelson interferometer for characterizing far-field VOF generated in the NIR regime.

**
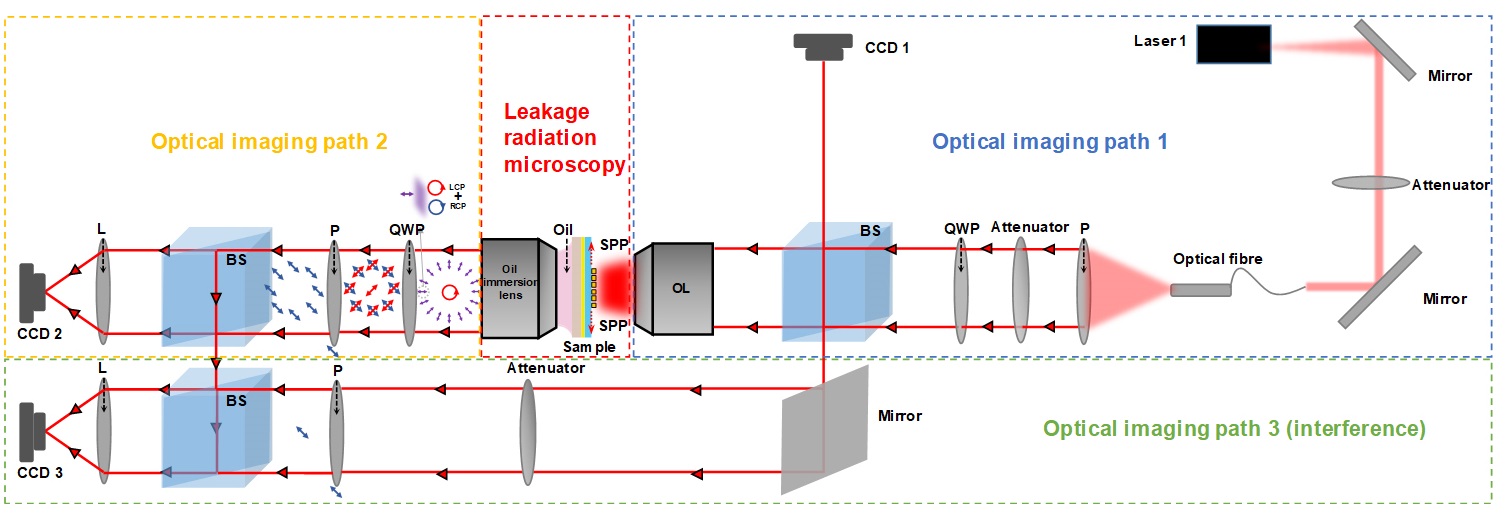
**

**Fig. S17** OIL leakage radiation microscopy system for SPP characterization at NIR regime

Section 9. Meta-devices designed according to Eq. (3) under two different illuminations


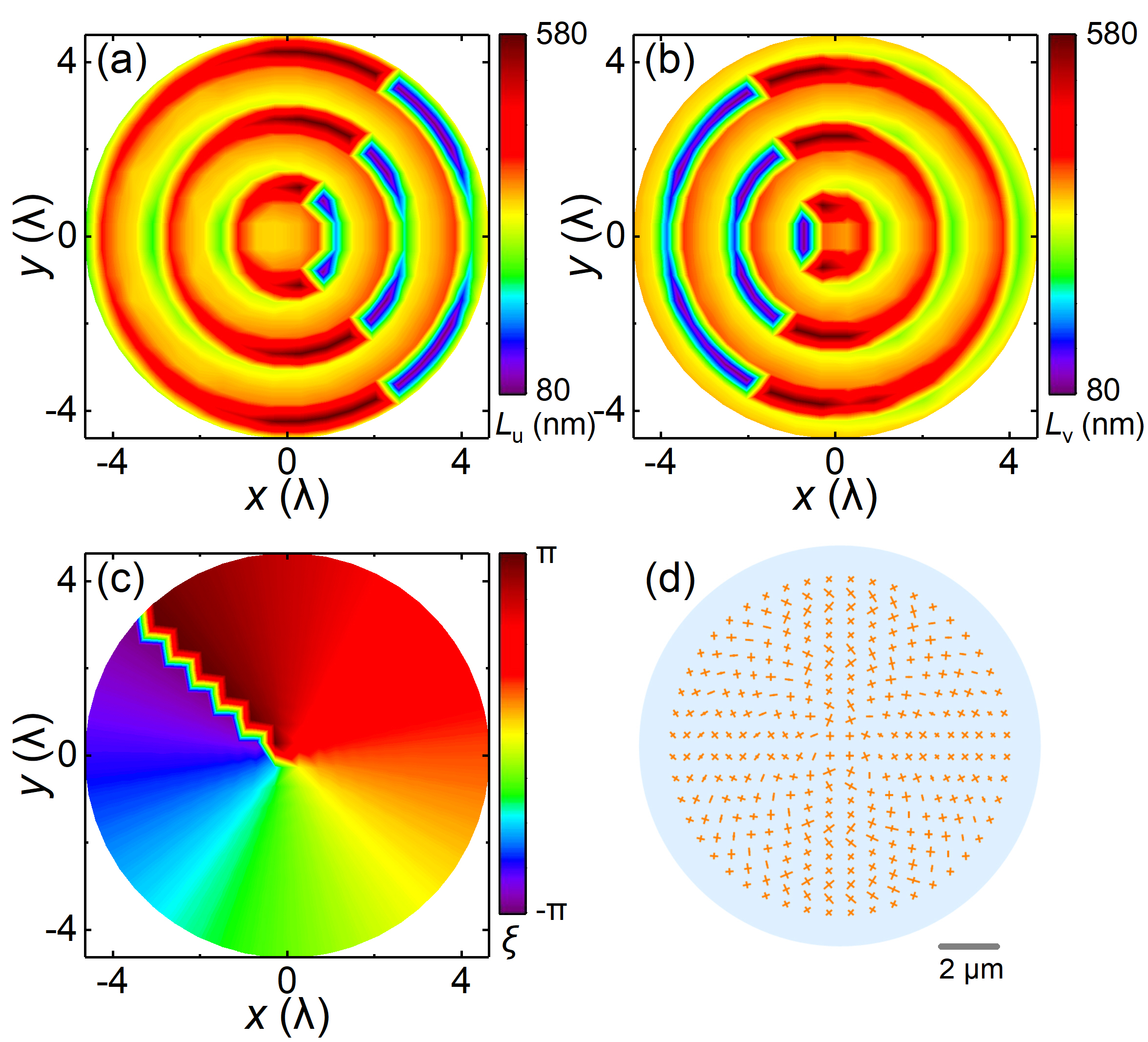


**Fig. S18** Structural details of meta-device A with properties given by Eq. (3) in the main text under the illumination of LCP light. Distribution of (a) $L_{u}$, (b) $L_{v}$, (c) $\xi$ and (d) layout of the Meta-device A.

We employ the strategy described in the main text to successfully design two meta-devices (denoted as A and B), both exhibiting properties given by Eq. (3) in the main text, but for LCP and elliptical polarization (EP) light illumination cases, respectively. Figures S18a-S18c and Figures S19a-S19c depict, respectively, the distributions of $L_{u}$, $L_{v}$ and $\xi$, while Fig. S18d and Fig. S19d illustrate the top-view structures of the designed meta-devices.


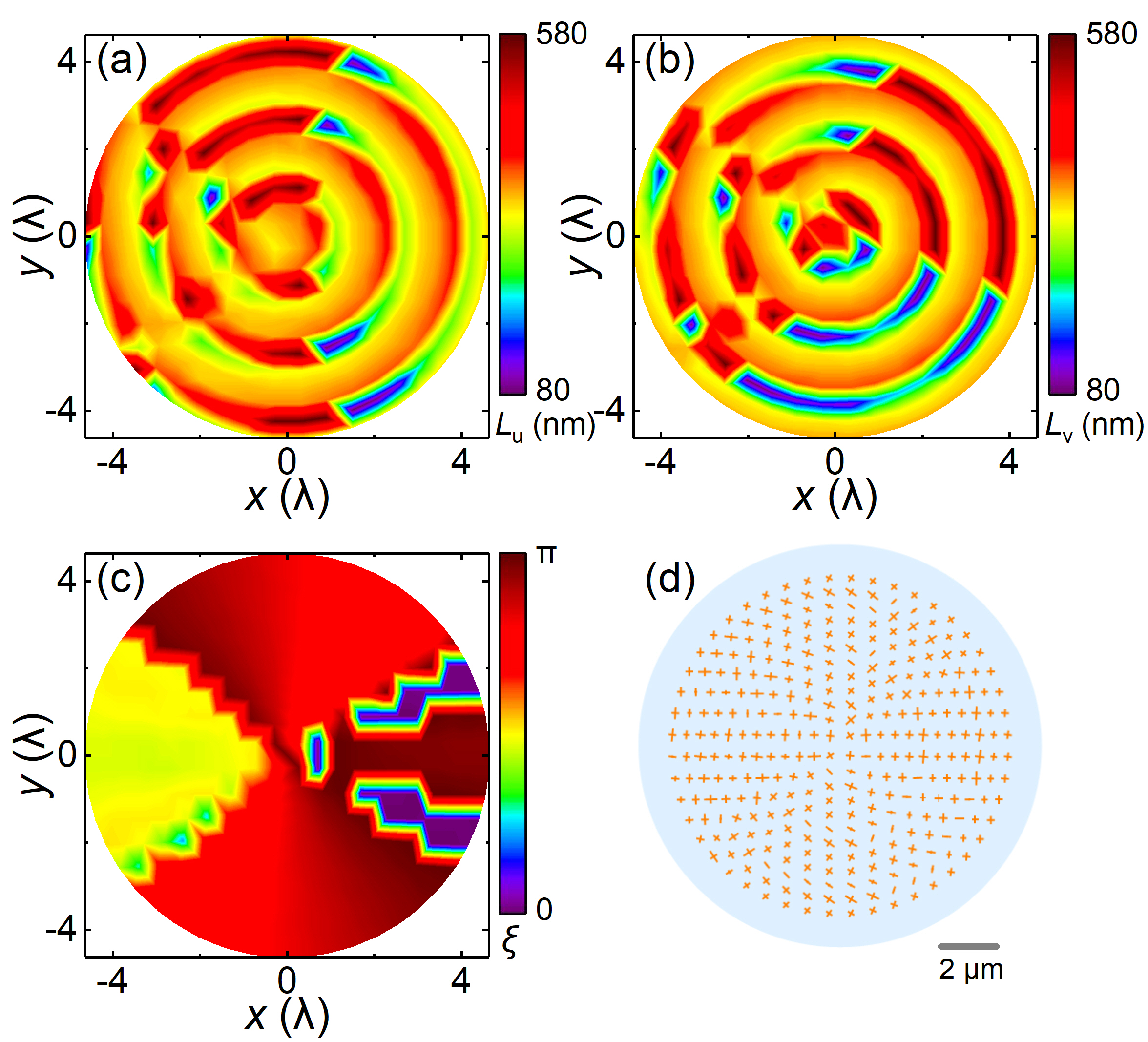


**Fig. S19** Structural details of meta-device B with properties given by Eq. (3) in the main text but under the illumination of an elliptically polarized light. Distribution of (a) $L_{u}$, (b) $L_{v}$, (c) $\xi$ and (d) layout for the Meta-device B.

We then further numerically studied the scattering properties of the designed meta-devices. Since the whole system is too large for performing full-wave simulations, we use the GF approach described in Sec. 2 in SI to study its scattering properties, with the material losses of realistic meta-atoms fully taken into account. Different from the ideal case (with all meta-atoms perfectly reflective) studied in Sec. 2.1, now our meta-atom at a point $\vec{r}$ is characterized by realistic complex reflection coefficients $r_{uu},\text{ }r_{vv}$ (but with $|r_{uu}|\text{，}|r_{vv}|<1$) given by full-wave simulations on the practically adopted lossy meta-atoms. In such a realistic case, current distribution adopted in the GF approach should be revised as

$\vec{J}(\vec{r})=J_{0}\delta(z)\left(\hat{e}_{+},\hat{e}_{-}\right)\left(\begin{matrix} e^{-i\frac{\Psi_{0}}{2}}\cos\frac{\Theta_{0}}{2}\frac{r_{uu}\left(\vec{r}\right)+r_{vv}\left(\vec{r}\right)}{2}+e^{-2i\xi\left(\vec{r}\right)}e^{i\frac{\Psi_{0}}{2}}\sin\frac{\Theta_{0}}{2}\frac{r_{uu}\left(\vec{r}\right)-r_{vv}\left(\vec{r}\right)}{2} \\ e^{+2i\xi\left(\vec{r}\right)}e^{-i\frac{\Psi_{0}}{2}}\cos\frac{\Theta_{0}}{2}\frac{r_{uu}\left(\vec{r}\right)-r_{vv}\left(\vec{r}\right)}{2}+e^{i\frac{\Psi_{0}}{2}}\sin\frac{\Theta_{0}}{2}\frac{r_{uu}\left(\vec{r}\right)+r_{vv}\left(\vec{r}\right)}{2} \end{matrix}\right)$(S16)

which automatically takes material losses into account by using realistic coefficients$r_{uu}\text{ }$and $r_{vv}$.

**
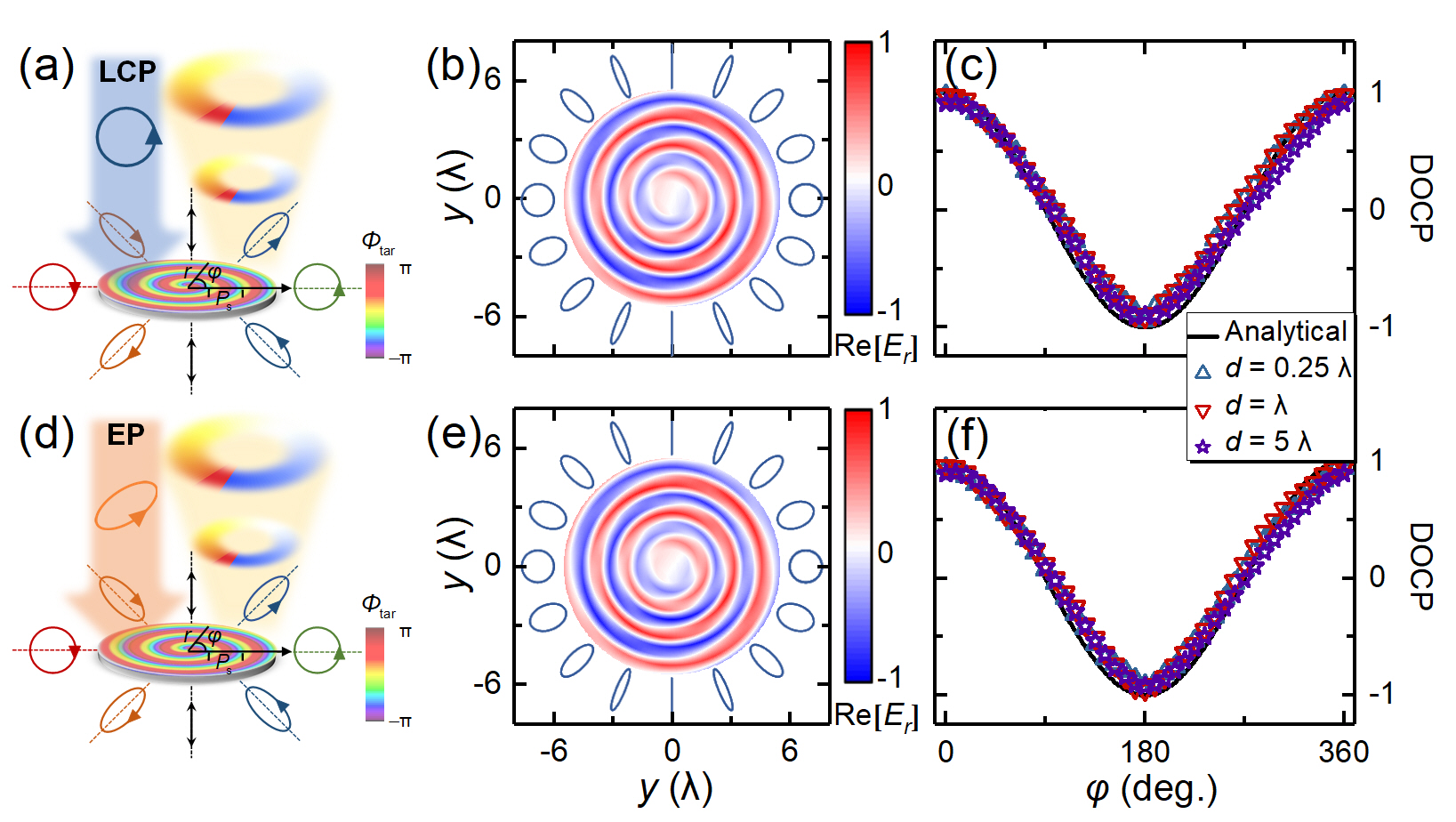
**

**Fig. S20** Schematics of light scatterings by (a) Meta-device A for LCP illumination case and (d) Meta-device B for EP illumination case ($\left(\Theta_{0},\Psi_{0}\right)=\left(2\pi/9,3\pi/8\right)$).$\mathrm{Re}[E_{r}]$ distribution for the reflected beams on an *xy*-plane locating at a distance $d=\lambda$ above (b) Meta-device A and (e) Meta-device B, calculated by the Green’s function approach with the surround ellipses and lines illustrating the polarization patterns at different angles. Degrees of circular polarization (DOCP) as functions of for the reflected waves on *xy*-planes at different distances (denoted by $d$) above (c) Meta-device A and (f) Meta-device B, calculated by the Green’s function approach. Solid lines in (c) and (f) are directly obtained according to Eq. (3).

We note that Eq. (S16) can go back to Eq. (S11) in the ideally lossless case for the LCP illumination cases with $r_{uu}=e^{i\Phi_{u}}$ and $r_{vv}=e^{i\Phi_{v}}$. Substituting Eq. (S16) into Eq. (S12), we obtain the corresponding scattering properties of the practically designed meta-devices and depict the results in Fig. S20. We note that calculations on the designed realistic structures, even with material losses taken into account, do not significantly change as compared to their ideal-model counterpart as shown in Fig. 2 of the main text. We thus demonstrate the feasibility of our design strategy described in the main text, which is established initially based on an ideal model.

**References**

1. Sun, S., He, Q., Xiao, S., Xu, Q., Li, X. & Zhou, L. Gradient-index meta-surfaces as a bridge linking propagating waves and surface waves. *Nature Mat.* **11**, 426-431 (2012).
2. Zhou, L., Huang, X. & Chan, C.T. A time-dependent Green’s function approach to study the transient phenomena in metamaterial lens focusing. *Photonics and Nanostructures: Fundamentals and Applications* **3**, 100-106 (2005).
3. Pors, A., Nielsen, M. G., Bernardin, T., Weeber, J. C. & Bozhevolnyi, S. I. Efficient unidirectional polarization-controlled excitation of surface plasmon polaritons. *Light Sci. Appl.* **3**, 197 (2014).
