# Supplementary figures and images for: Efficient generation of complex vectorial optical fields with metasurfaces
Source: Light Sci Appl. 2021 Mar 31;10:67. doi: 10.1038/s41377-021-00504-x (PMC8012391; doi:10.1038/s41377-021-00504-x)

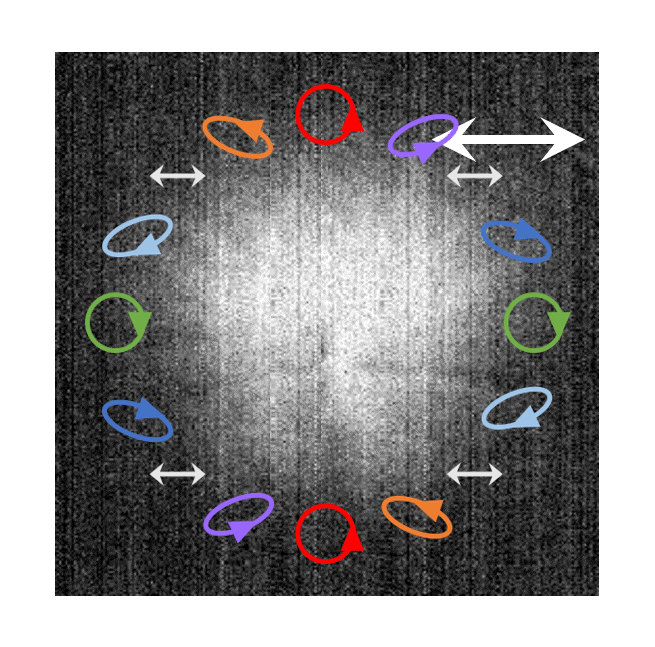

Supplement: Supplementary file 2 — Movie 1 [file 41377_2021_504_MOESM2_ESM.gif]
